# Enhancer reprogramming: critical roles in cancer and promising therapeutic strategies

**DOI:** 10.1038/s41420-025-02366-3

**Published:** 2025-03-03

**Authors:** Jinshou Yang, Feihan Zhou, Xiyuan Luo, Yuan Fang, Xing Wang, Xiaohong Liu, Ruiling Xiao, Decheng Jiang, Yuemeng Tang, Gang Yang, Lei You, Yupei Zhao

**Affiliations:** 1 Department of General Surgery, Peking Union Medical College Hospital, Peking Union Medical College, Chinese Academy of Medical Sciences, Beijing, PR China; 2 Key Laboratory of Research in Pancreatic Tumor, Chinese Academy of Medical Sciences, Beijing, PR China; 3 National Science and Technology Key Infrastructure on Translational Medicine in Peking Union Medical College Hospital, Beijing, PR China

**Keywords:** Cancer genomics, Cell biology, Epigenetics, Gene regulation

## Abstract

Transcriptional dysregulation is a hallmark of cancer initiation and progression, driven by genetic and epigenetic alterations. Enhancer reprogramming has emerged as a pivotal driver of carcinogenesis, with cancer cells often relying on aberrant transcriptional programs. The advent of high-throughput sequencing technologies has provided critical insights into enhancer reprogramming events and their role in malignancy. While targeting enhancers presents a promising therapeutic strategy, significant challenges remain. These include the off-target effects of enhancer-targeting technologies, the complexity and redundancy of enhancer networks, and the dynamic nature of enhancer reprogramming, which may contribute to therapeutic resistance. This review comprehensively encapsulates the structural attributes of enhancers, delineates the mechanisms underlying their dysregulation in malignant transformation, and evaluates the therapeutic opportunities and limitations associated with targeting enhancers in cancer.

## Facts

- Enhancers play a central role in the transcriptional mechanism of cancer, and various intracellular and extracellular factors(such as transcription factors, cytokines, and multiple signaling pathways) can act on them and drive the formation of core regulatory circuits—a highly integrated regulatory network composed of master transcription factors, their associated enhancers, and target genes.
- Enhancer reprogramming in cancer not only unlocks the phenotypic plasticity of cancer cells, enabling traits such as proliferation, drug resistance, and metastasis but also contributes to the reshaping of the tumor microenvironment, driving cancer progression through more complex mechanisms.
- Based on previous research, this article proposes different potential targeting therapeutic strategies, especially based on the unique mechanism of enhancers regulating the tumor microenvironment discovered recently, as well as comprehensive therapy. For instance, the combined application of chemotherapy, immunotherapy, and enhancer-targeted therapies, aiming to leverage their complementary mechanisms to overcome the limitations of single treatments and improve therapeutic outcomes (e.g. NCT06393361, NCT06563778).

## Open questions

- What key molecules or signaling pathways determine the selective activation or silencing of enhancers in cancer cells?
- What advanced techniques and models can be utilized to capture dynamic enhancer responses during tumor progression and treatment, and how can these insights guide therapeutic strategies?
- Based on previous research findings, we have provided some alternative targeted treatment strategies. Recent studies have explored combination strategies involving metabolic pathway inhibitors, like the use of purine synthesis inhibitors in MLL3/4-mutant cancers, and have demonstrated synergistic effects in preclinical models. Whether these comprehensive treatment strategies are feasible and can they improve patient prognosis?

## Introduction

Transcription initiation is a key step in the regulation of gene expression. In eukaryotic cells, transcription begins with the binding of RNA polymerase to the promoter, which is regulated by transcription factors (TFs) [1]. Enhancers are DNA sequences located upstream or downstream of gene promoters, typically far from the promoter region, and are rich in TF binding sites (TFBS). They significantly increase gene expression in ways that are independent of direction and distance [2]. Annotation of enhancers on a genome-wide scale has been achieved through various chromatin immunoprecipitation (ChIP) techniques (e.g., ChIP-sequencing [ChIP-seq]) and chromatin accessibility analyses, including DNase-seq and the assay for transposase-accessible chromatin using sequencing (ATAC-seq). These methods were prominently utilized in the Encyclopedia of DNA Elements (ENCODE) project to define different classes of regulatory elements [3–5]. Additionally, high-throughput chromosome conformation capture technologies have identified the direct interaction between enhancers and promoters within the three-dimensional (3D) chromatin architecture, highlighting the functional relevance of spatial genome organization in enhancer activity [6].

Higher-order assembly of TFs—the cooperative and hierarchical interactions between TFs, co-activators, and chromatin remodelers—on enhancers is tightly regulated to ensure tissue-specific gene expression and cellular homeostasis in normal cells [7–9]. However, in cancerous cells, genetic mutations in TFs or chromatin regulators disrupt this balance, leading to aberrant enhancer activity [10–12]. Recent studies highlight the critical role of somatic mutations in enhancer dysfunction, revealing how these mutations perturb enhancer-promoter interactions [13, 14]. Mutations in epigenetic regulators like TET2 and MLL4 have been shown to disrupt enhancer-associated chromatin modifications, rebalancing key transcriptional programs in cancer [15, 16]. Moreover, structural variants, including duplications or translocations, can relocate enhancers near oncogenes, further amplifying their expression and accelerating tumor progression [17]. These insights establish a direct link between genetic mutations and enhancer dysfunction, underscoring their importance in the pathogenesis of cancer.

The deregulation of genes is a major feature of cancer, involving both genetic mutations and non-coding epigenetic reprogramming [18]. An increasing number of analyses of cancer genomes and epigenomes have shown that enhancers play a crucial role in driving the gene expression regulatory networks in cancer [19, 20]. Cancer cells hijack enhancers as platforms to integrate intracellular and extracellular signals, binding with key TFs to establish self-circulating core transcriptional axes [21]. This process dynamically confers new phenotypes to cancer cells. Cancer cells become addicted to the high transcriptional output driven by enhancers; thus, the vulnerabilities of cancer cells become potential therapeutic targets [20]. However, the specificity of targeting enhancers remains a challenge, as off-target effects or disruptions to normal enhancer activity could lead to adverse outcomes [22]. Addressing these challenges requires a deeper understanding of the molecular underpinnings of enhancer dysfunction and its links to genetic mutations, as well as the development of precision therapeutic strategies to selectively target enhancer-driven transcriptional addictions.

## Structural and functional characteristics of enhancer

**Fig. 1:**
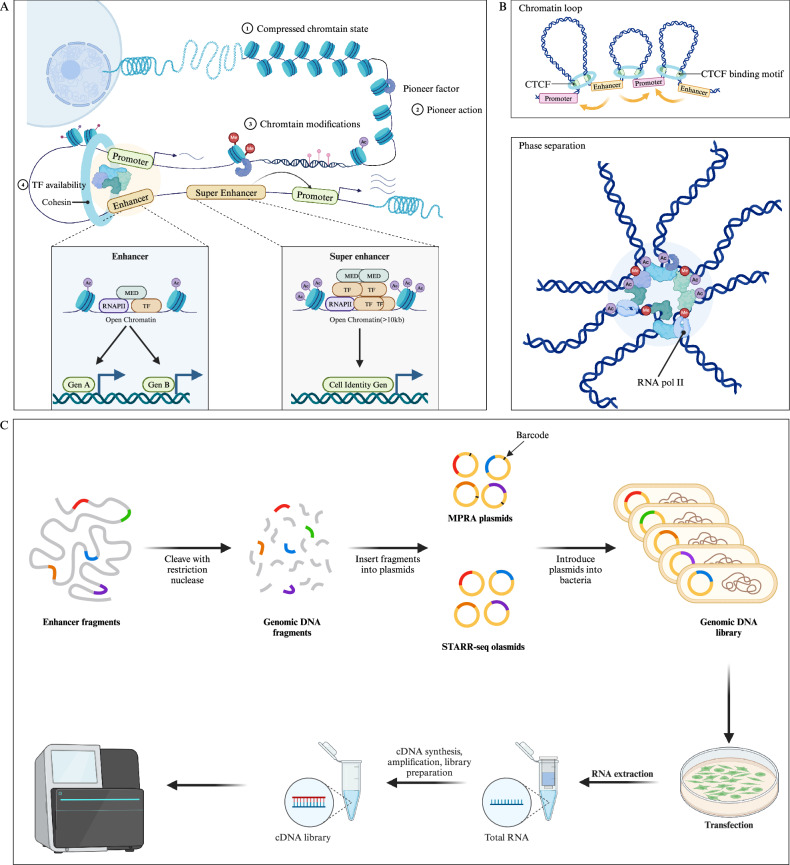
Structural and functional characteristics of enhancer. **A** Pioneer factors bind to and activate closed chromatin by mediating chromatin remodeling, making it accessible for other TFs. In contrast, open chromatin structures do not require this mechanism, as TFs can directly access these open enhancers. Pioneer factors recruit epigenetic modifiers to alter the local chromatin environment (such as histone methylation, acetylation, and DNA methylation), further collaborating with other TFs to activate transcription. The figure illustrates a typical enhancer and SE, with SE enriched in TFs and histone modifications (e.g., histone acetylation), driving the expression of cell identity genes. **B** Chromatin loop (up) and phase separation (down) model of enhancers. In the chromatin loop model, CTCF and cohesins regulate the 3D structure of chromatin through a loop extrusion mechanism, promoting enhancer-promoter interactions. In the phase separation model, the interaction between enhancers, high-density TFs and chromatin modifications forms liquid-like condensates, further modulating gene expression. **C** High-throughput detection of enhancer activity of DNA fragments using MRPA and STARR-seq methods. (Created with BioRender.com).

Enhancers are defined as non-coding DNA sequences capable of promoting the expression of target genes. It has been demonstrated that enhancers play crucial roles in the development and diseases [23]. In many cases, enhancers are located at significant distances from their target genes (even skipping over unrelated genes to interact with the target promoter) and are regulated by tissue-specific TFs and cofactors, such as p300/CREB-binding protein (p300/CBP), mediator (MED), and switch/sucrose non-fermentable (SWI/SNF) [24]. Through these long-range interactions, enhancers can precisely control the expression of specific genes in a temporal and spatial manner (Fig. 1) [25].

### Enhancer and TFs

It is well established that the activity of enhancers is regulated by numerous transcription factors (TFs) [26]. Currently, TFs commonly found at enhancers are broadly categorized into two types: tissue-specific TFs and general TFs enhancers [27]. For example, p300/CBP, a histone acetyltransferase (HAT), is enriched at enhancers and facilitates chromatin accessibility, serving as a hallmark of enhancer activity [28]. MED1, a core component of the Mediator complex, acts as a bridge between enhancers and promoters by recruiting RNA polymerase II to initiate transcription (Fig. 1A) [29]. In various species and tissues, the transcriptional activity of enhancers differs significantly, driven by lineage-specific transcription factors (TFs) that are uniquely expressed in specific cells or tissues. For instance, FOXA1 and FOXA2 were initially identified as tissue-specific factors during foregut endoderm development [30, 31]. Interestingly, some tissue-specific TFs also serve as pioneer factors under certain conditions, and not only function within specific tissues but can also bind to closed chromatin regions and remodel them into an open state, embodying the hallmark characteristics of pioneer factors [32]. This “dual role” underscores their unique position in gene regulation.

FOXA1 and FOXA2 exhibit classic pioneer activity by binding to closed chromatin regions and opening them [33]. Their “winged-helix” DNA-binding domain enables them to penetrate nucleosomal DNA and expose chromatin regions, facilitating the binding and activation of downstream tissue-specific factors such as HNF4α in the liver and Pdx1 in the pancreas [34, 35]. Within these open chromatin regions, pioneer factors and tissue-specific TFs interact synergistically, stabilizing each other’s binding and enhancing their regulatory effects. For instance, FOXA2, by interacting with JUN, activates lineage plasticity enhancers, driving prostate cancer cells to transition from AR-dependence to a multilineage state [36]. In some cases, pioneer factors primarily open chromatin, while tissue-specific TFs determine which genes are ultimately activated. For example, SOX2 acts as a pioneer factor across various cell types, but its downstream targets are determined by co-binding with tissue-specific factors, such as OCT4 in embryonic stem cells [37, 38]. This combinatorial mechanism endows enhancers with high specificity, allowing the same pioneer factor to work with distinct tissue-specific TFs to activate unique gene expression programs in different cell types.

However, the precise mechanisms by which pioneer factors activate closed chromatin remain elusive. Some studies suggest that pioneer factors facilitate chromatin opening by inducing nucleosome repositioning [39]. For example, OCT4 binds nucleosomes through two DNA-binding domains: one anchor at the nucleosome entry site, while the other engages distal DNA, bending and exposing the DNA for nucleosome repositioning. Interestingly, OCT4 can lock DNA into specific conformations while exposing binding sites for SOX2. Moreover, H3K27ac-induced DNA sliding enhances OCT4’s multi-site binding and stabilizes the recruitment of other TFs [39].

Additionally, intrinsically disordered regions (IDRs) in pioneer factors contribute to chromatin remodeling by forming condensates or interacting with chromatin [40, 41]. For example, the IDRs of PU.1 facilitate weak interactions with chromatin components such as histone tails or non-specific DNA sequences, driving structural changes in local chromatin [40]. Similarly, the N- and C-terminal IDRs of FOXA1 enable the formation of sub-micrometer biomolecular condensates, effectively loosening compact chromatin to enhance accessibility [41]. Recent studies further indicate that the activation of closed chromatin by pioneer factors involves multi-step modifications and structural adjustments [42]. For instance, before cell division, PAX7 binds chromatin and recruits the H3K9me2 demethylase KDM1A (LSD1) and MLL, gradually converting chromatin from a repressive to a potentially active state. During cell division, enhancers dissociate from lamin B structures at the nuclear periphery, while PAX7 simultaneously recruits the SWI–SNF chromatin remodeling complex and p300, further opening enhancer regions to facilitate binding by transcription factors and co-activators [42].

### Characterizing the functional syntax of enhancers

Current research highlights the critical role of histone modifications in enhancer identification and function [43]. Modifications such as H3K4me1 and H3K27ac are widely recognized as markers of active enhancers, aiding in their identification [44]. Furthermore, recent studies have revealed additional histone marks associated with active enhancers, including H3K18la [45], H4K16ac [46], H3K9ac [47, 48], H2BNTac [49], crotonylation [50], and β-hydroxybutyrylation (e.g., H3K56bhb) [51]. However, while the distribution of histone modifications provides valuable locational cues, the levels of these marks alone are insufficient to predict the functional activity of enhancers.

Enhancer function involves a complex “syntax” that integrates multiple elements beyond histone marks, including specific DNA sequences, TFBS, chromatin accessibility, and environmental cues [24, 52–54]. Thus, while histone modifications offer important insights into enhancer identification, a comprehensive understanding of enhancer regulation requires moving beyond modification patterns to explore the intricate molecular grammar that governs their activity.

Traditional methods for assessing enhancer activity in DNA sequences rely on reporter gene assays, such as luciferase assays, which evaluate the transcriptional activity of inserted sequences by measuring luminescence intensity [55]. However, these approaches are primarily used for validation and are not suitable for high-throughput screening. High-throughput techniques such as self-transcribing active regulatory region sequencing (STARR-seq) and massively parallel reporter assay (MPRA) have enabled genome-wide detection of potential enhancer activity (Fig. 1C) [56–58].

Moreover, machine learning models trained on these high-throughput sequencing datasets have proven effective in predicting active enhancers and TFBS (Table 1) [59, 60]. The combined application of ChIP-seq or ATAC-seq with STARR-seq (e.g., ChIP-STARR-seq and ATAC-STARR-seq) further advances the identification and functional validation of enhancers at a genome-wide scale, providing a powerful framework for elucidating regulatory elements and their activity [44, 61, 62].

**Table. 1:** Parse enhancer syntax with machine learning algorithms.

Classification	Algorithms	Description	References
Support Vector Machine (SVM)	ChromaGenSVM	Predicting enhancers based on the optimal combination of histone epigenetic markers.	[350]
	Gkm-SVM	Use SVM to identify enhancer regions by calculating nucleotide level importance scores.	[351]
	EnhancerFinder	Multi-kernel learning algorithm based on support vector machine for predicting developmental enhancers.	[352]
Random Forest (RF)	RFECS	Based on the random forest algorithm, predict enhancers by analyzing chromatin state data.	[353]
	DRAF	Used to predict TFBS.	[354]
Convolutional Neural Network (CNN)	DeepEnhancer	Predicting enhancers through deep convolutional neural networks and processing variable length sequences in the FANTOM5 dataset.	[355]
	DeepBind	Used to predict TFBS.	[356]
	DESSO	Used to predict regulatory motifs from human ChIP-seq data.	[357]
	Basset	Predicting chromatin accessibility by classifying features in the sequence.	[358]
	DeepSTARR	Used to predict enhancers with development activities.	[359]
	BPNet	Predicting transcription factor binding profiles at single nucleotide resolution using convolutional neural networks.	[360]
	Enformer	Predicting enhancer-promoter interactions from DNA sequences.	[60]
	iEnhancer-ECNN	Using the ensemble of CNN to identify enhancers and their intensities.	[361]
	GhmCN	Predicting gene expression status 5hmC signaling and prioritizing potential enhancers	[362]
	DeepCAPE	Predicting enhancers by integrating DNA sequences and DNase-seq data.	[363]
	CoNSEPT	Used to predict enhancer function under different conditions, such as cell type and experimental conditions.	[364]
	DEEPSEN	Integrating 36 features for predicting super-enhancers.	[365]
	ES-ARCNN	Expand the previously identified enhancer dataset through data augmentation techniques (i.e. reverse complement and shift).	[366]
Hybrid architecture	DanQ	Combining CNN and bidirectional recurrent neural networks for predicting the features of regulatory regions.	[367]
	BiRen	Combining CNN and bidirectional recurrent neural networks for predicting enhancers.	[368]
	DeepATT	A mixed-class attention neural network for identifying functional effects of DNA sequences.	[369]
	Enhancer-IF	Integrating RF, extremely random tree, multilayer perceptron, SVM, and extreme gradient enhancement to enhance model robustness.	[370]
	SEMet	Predicting enhancers associated with metastasis and prognosis of pancreatic cancer by SE landscape.	[371]
	CAPReSE	Using CNN and XGBoost to identify specific structural variation (SV)-mediated aberrant chromatin contacts in cancer genomes, with particular application in colorectal cancer.	[107]

In cancer research, by integrating eCLIP, Hi-C, and genome-wide STARR-seq, a recent study utilized ENCODE data to annotate cancer-related cell types and help construct regulatory networks that include TFs and RNA-binding proteins. This effectively describes the cell state trajectories and regulatory network reorganization during cancer transformation [63]. Regulatory single nucleotide polymorphisms associated with cancer risk were systematically identified, revealing the regulatory mechanisms of these variants on gene expression. For example, rs11055880 is associated with breast cancer by regulating the expression of ATF7IP [64]. Besides, a study that integrated DNase-seq, ChIP-seq, global run-on sequencing, STARR-seq, RNA-seq, Hi-C, and Chromatin Interaction Analysis with Paired-End-Tag (ChIA-PET) data from five cancer cell lines identified a new class of autonomous and dual SEs. These SEs regulate the transcription of highly expressed genes through long-range chromatin interactions and promote the survival of cancer cells [65]. The integration of high-throughput sequencing technologies with CRISPR can facilitate the identification of non-coding genetic variants with significant regulatory effects in cancer and link these regulatory perturbations to differences in therapeutic sensitivity. For example, in pediatric acute lymphoblastic leukemia, the combination of MPRA and CRISPR identified rs1247117 as a key variant influencing vincristine sensitivity [66].

It is important to note that both reporter assays, such as STARR-seq, and MPRA are ectopic detection methods that cannot fully recapitulate the native chromatin environment [57, 67]. Additionally, enhancer activity varies significantly across cell types, introducing inherent limitations, particularly in studies involving clinical samples [68]. A potential solution lies in leveraging single-cell ATAC-seq and CUT&Tag sequencing to gain a clearer understanding of the cell-specific nature of enhancers [69, 70]. Besides, the recent development of a single-cell MRPA method may also help address this issue [71]. These approaches preserve the original chromatin architecture and the influence of environmental factors on enhancer function. When combined with single-cell transcriptomics, they can provide deeper insights into enhancer activity within its native context.

TFs play a critical role in regulating enhancer activity. Recent studies suggest that enhancer function is not solely determined by TF binding strength but also by dynamic parameters such as TF clustering frequency, periodicity, and stability [72]. Supporting this view, enhancer function remains stable through cooperative and combinatorial TF interactions, even when evolutionary changes occur in sequences or individual TF binding sites [73]. During somatic cell reprogramming to pluripotent stem cells, the OSKM factors (Oct4, Sox2, Klf4, and c-Myc) initially bind to somatic cell enhancers, recruiting histone deacetylases (e.g., HDAC1) and redistributing somatic-specific TFs (e.g., Fra1), effectively silencing somatic enhancers. As reprogramming progresses, OSKM factors relocate to pluripotency-associated enhancers, depositing H3K27ac marks and recruiting key pluripotency TFs (e.g., Nanog and Esrrb) to support the expression of pluripotency genes [7].

In cancer, TFs such as YAP/TAZ/TEAD co-occupy enhancers with AP-1. The tumor-promoting effects of YAP/TAZ are dependent on AP-1, and AP-1 inactivation significantly impairs YAP/TAZ-driven tumor cell proliferation [74]. Additionally, FOXC1, typically absent in normal hematopoiesis, is highly expressed in certain acute myeloid leukemia (AML) subtypes. FOXC1 recruits transcriptional repressors RUNX1 and TLE3 to enhancers regulating monocyte differentiation, thereby blocking AML cell differentiation. FOXC1 deletion disrupts the binding of RUNX1 and TLE3, increasing the occupancy of differentiation-related TF CEBPA at these enhancers [75]. Similarly, SOX9, a key TF driving the transition from epidermal stem cells to hair follicle stem cells, transiently activates and redistributes chromatin regulators such as SWI/SNF and MLL3/4 to hair follicle enhancers, silencing epidermal genes. However, prolonged SOX9 activation selectively engages tumor-associated enhancers, leading to the activation of the Wnt/β-catenin signaling pathway [76]. These findings highlight the critical role of higher-order TF assemblies in the dynamic regulation of enhancers and reveal distinct TF dynamics between normal and cancer cells.

Moreover, current research suggests that enhancer RNA (eRNA) transcription is a hallmark of active enhancers [77, 78] Knockdown of eRNAs disrupts TF binding to enhancers, such as c-Jun, NF-κB, and YY1. eRNAs also interact with MED1 and RNAPII to facilitate enhancer-promoter looping. A recent study demonstrated that eRNAs directly bind the AT-hook domain of Brg1 to recruit the SWI/SNF chromatin remodeling complex to specific enhancers, simultaneously enhancing the recruitment and activation of co-activators such as MLL3/4, MED1, and p300/CBP [52]. Additionally, m6A-modified eRNAs can recruit the m6A reader protein YTHDC1, forming droplet-like condensates at enhancers. These condensates promote the clustering of co-activators like BRD4, thereby increasing enhancer activity and gene transcription [79]. These findings indicate that eRNAs are critical regulators within gene regulatory networks, playing an essential role in maintaining and modulating the dynamic balance of gene expression, although predicting enhancer activity directly from eRNAs remains challenging based on current knowledge.

### The paradigm of enhancer function

The transcriptional information mediated by enhancers is transmitted through enhancer-promoter (E-P) interactions [80]. High-resolution imaging techniques have revealed complex E-P interaction networks and proposed several major E-P interaction models, such as the chromatin loop model mediated by CCCTC-binding factor (CTCF) and cohesin, phase separation, the scanning model, and the tracking model (Fig. 1B) [54, 81].

The 3D structure of the genome is organized into basic units termed topologically associating domains (TADs), maintained by boundary elements such as CTCF and cohesion. DNA sequences within these regions interact more frequently between them than with sequences in adjacent TADs [82]. In the chromatin loop model, the cohesin complex extrudes DNA loops, bringing distant enhancers and promoters into close proximity for functional communication. Of note, CTCF acts as an anchor to prevent incorrect loop formation and enhancer off-target effects [83, 84]. Despite significant support for this model, some evidence challenges its usefulness. For example, current loop structures suggest that cohesin does not affect the expression of most genes, except for those associated with SEs. Additionally, gene expression and repression coexist within some TADs. Furthermore, genome-wide deletion of CTCF does not significantly alter gene expression levels, as observed in studies on SOX2 and SOX9 [81, 85, 86].

The phase separation model is a newer theory explaining enhancer function [87]. This model is based on the concept of biomolecular condensation and phase separation, suggesting that enhancers regulate gene expression by forming droplet-like condensates [87]. For example, general TFs (bromodomain-containing protein 4 [BRD4] and MED1) can concentrate the transcription machinery at enhancers through phase separation, thereby forming condensates [88]. Dysregulation of condensates is considered a hidden driver in cancer pathology. Certain proteins in cancer cells (e.g., Yes1 associated transcriptional regulator [YAP], homeobox B8 [HOXB8], and FOS like 1 [FOSL1]) promote condensate formation, thus directly enhancing oncogene expression and cell proliferation. Additionally, condensate dysregulation can alter the chromatin structure, impacting normal gene expression and regulation [89, 90].

### SEs

Through an in-depth exploration of the genome, researchers have identified enhancers exhibiting unique characteristics, termed SEs, which extend across larger genomic regions [91]. In comparison to conventional enhancers, SEs exhibit several distinctive features: (1) they span a larger genomic region, typically >10 kb; (2) they display significantly augmented histone modifications, notably H3K27ac enrichment; (3) they are intricately associated with specific cell types or states, exerting robust regulation of gene expression linked to cell characteristics such as differentiation and identity maintenance; (4) they are closely implicated in the onset and progression of diverse diseases, particularly cancer; (5) they govern lineage-specific TFs, including SOX2, KLF5, FOXA1, and FOXA2; and (6) they are more susceptible to intervention. This characterization underscores the pivotal role of SEs in gene regulation, cell identity, and disease pathology, presenting them as promising targets for intervention (Fig. 1A) [92–94].

## Enhancer reprogramming in cancer

Enhancer reprogramming refers to the dynamic changes in chromatin state and enhancer activity during cell fate determination, differentiation processes, or disease states [95–97]. Numerous factors act on enhancers through complex interactions and regulatory networks, leading to widespread changes in gene expression that promote the development and progression of cancer [98–100]. Understanding these mechanisms is crucial for revealing the fundamental principles of gene expression regulation, as well as for the diagnosis and treatment of cancer. Based on the structural and functional characteristics of enhancers, the factors leading to enhancer reprogramming can be divided into two main categories, namely genetic mutations and epigenetic remodeling.

### Somatic mutations

**Fig. 2:**
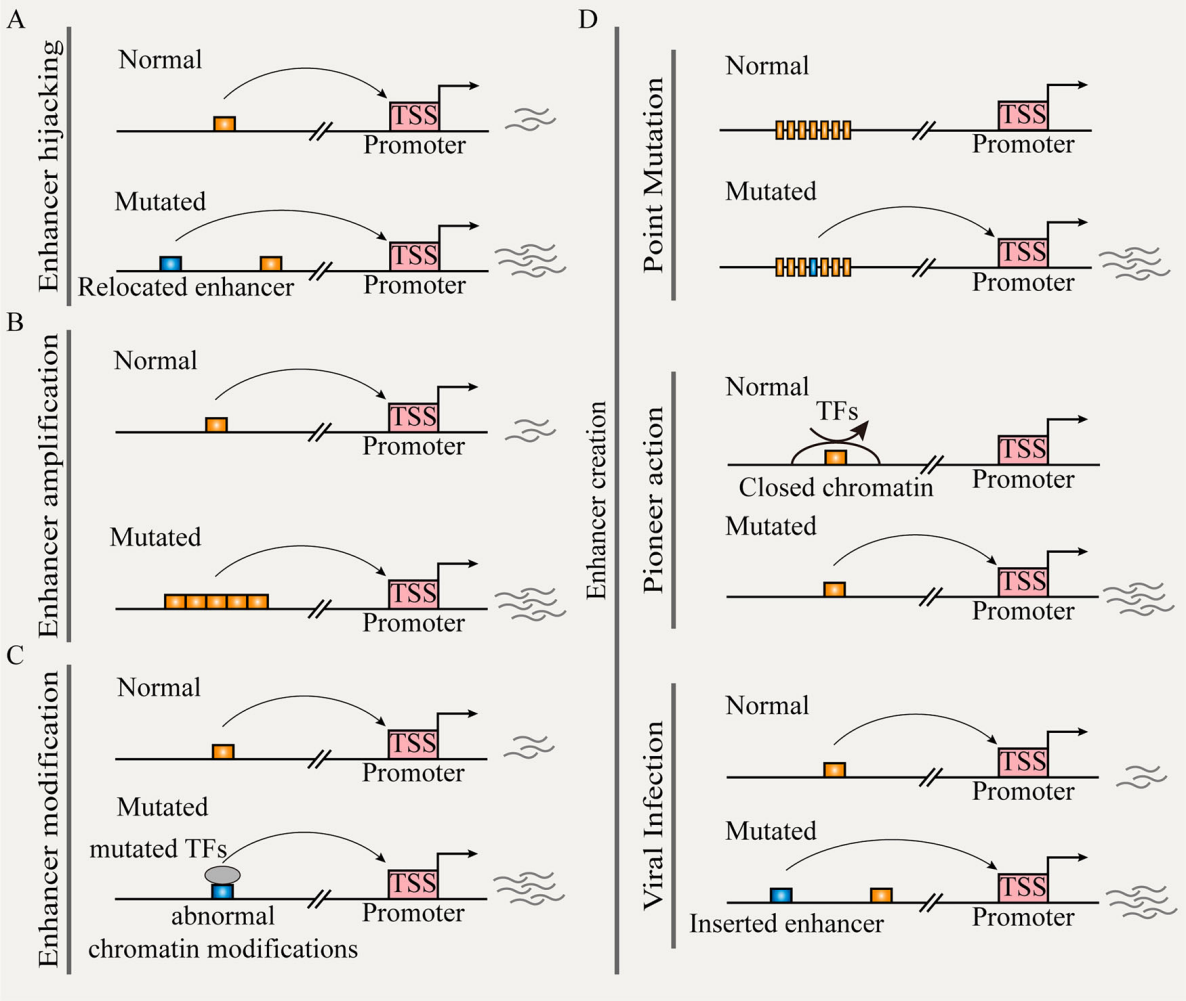
Somatic mutation and enhancer reprogramming. **A** Chromosomal structural variation leads to the repositioning of distal enhancers to target promoters. **B** Non-coding copy-number variation leads to enhancer function amplification. **C** Mutated TFs cause enhancer reprogramming. **D** Random mutation, pioneer action, and viral infection can create de novo enhancers.

Recent research has revealed the reprogramming of enhancer functions by genetic mutations through different pathways, leading to abnormal gene expression and disease [20]. We explore several mechanisms (e.g., enhancer hijacking, enhancer creation, and enhancer modulation), illustrating their specific roles in gene regulation and discussing their impact on cancer (Fig. 2).

#### Enhancer hijacking

Chromosomal structural variations (SVs) (e.g., inversions, translocations, and fusions) directly relocate the ectopic enhancer to the vicinity of the target gene; this process is referred to as ‘enhancer hijacking’ (Fig. 2A) [17, 101, 102]. This event occurs frequently in hematologic malignancies. During acute myeloid leukemia (AML), there are recurrent and subtype-specific alterations in A/B compartments, TADs, and chromatin loops. These SVs lead to widespread enhancer-hijacking and silencer-hijacking events, thereby promoting the progression of AML [17]. In AML cells with inv (3)/*t* (3:3), for example, the original enhancer of GATA2 located near the ribophorin I (RPN1) gene relocated to the EVI1 locus and formed SE. This resulted in single allelic expression of GATA2 and abnormal activation of EVI1 [103]. Previous studies have established a comprehensive computational framework for predicting enhancer hijacking based on Hi-C data [104]. Recently, the integration of multi-modal analysis methods (e.g., whole-genome sequencing, Hi-C, RNA-seq, ChIP-seq, and ATAC-seq) has provided new opportunities to deeply understand the impact of SVs on gene regulation through chromatin interactions. For instance, in certain pediatric leukemia subtypes, the recurrent *t*(5;14) translocation results in enhancer hijacking of BCL11 transcription factor B (BCL11B), specifically activating the T cell leukemia homeobox 3 (TLX3) gene [105]. A recent study analyzing 92 cancer cell lines and clinical samples revealed that SVs lead to the formation of numerous fused TADs. This effect leads to the accumulation of a greater number of highly active enhancers and is closely associated with the activation of oncogenes, such as MYC, telomerase reverse transcriptase (TERT), and cyclin D1 (CCND1). Besides, using a deep learning-based Activity-By-Contact (ABC) model, researchers can predict which SVs are likely to activate oncogenes [106]. The machine learning tool CAPReSE has been developed for chromatin anomaly pattern recognition and size estimation. This tool accurately identifies specific SV-mediated aberrant chromatin contacts in cancer genomes. In colorectal cancer, CAPReSE identified numerous recurrent enhancer-hijacking events mediated by SVs. These events involve cell cycle and DNA processing and may play important roles in tumor progression and chemotherapy resistance [107]. Additionally, by integrating somatic copy-number variations, gene expression data, and TAD information, the cis-Expression Structural Alteration Mapping (CESAM) systematically identifies enhancer-hijacking events in pan-cancer genomes. For instance, in colorectal cancer, recurrent tandem duplications intersecting with TAD boundaries result in the formation of new 3D contact domains, encompassing insulin-like growth factor 2 (IGF2) and a lineage-specific SE, leading to high-level gene activation [108].

#### Enhancer creation

We define the “enhancer creation” as the process through which new regulatory elements emerge in the genome by various genetic and epigenetic mechanisms. For example, during the development of pancreatic cancer, transient precursor cells of pancreatic cancer will appear in pancreatitis, characterized by the formation of transient enhancer networks mediated by KRAS mutation. In this process, KRAS mutation stabilizes the key oncogenic activator protein 1 (AP-1) TFs (JUNB and FOSL1), locking the progenitor state to initiate tumor development [109].

Random mutations drive enhancer reprogramming by altering TFBS, rewiring promoter-enhancer connections, and modifying epigenetic marks (Fig. 2D). Enhancer activity typically relies on specific TFBS, and random mutations within enhancer regions can either create new binding sites or disrupt existing ones, leading to changes in regulatory function. For instance, in T-ALL, the insertion upstream of the TAL bHLH transcription factor 1, erythroid differentiation factor (TAL1) oncogene introduces novel MYB binding sites, resulting in the formation of SE that drives TAL1 [110]. In neuroblastoma, single nucleotide variants in the SE within the first intron of LIM domain only 1 (LMO1) eliminate the binding motif of GATA and cause LMO1 dysfunction [111]. Consistent with this, an integrated study of whole-genome and epigenome data in lung cancer revealed that enhancer mutations can create new binding sites for oncogenic TFs such as MYC or NF-κB. Conversely, other enhancer mutations disrupt binding sites for tumor-suppressive TFs, thereby weakening the transcriptional repression of oncogenes. These mutations also influence the epigenetic modifications of enhancers, leading to either increased or decreased H3K27ac levels [13]. Furthermore, random mutations can alter the 3D structure of chromatin, thereby modifying spatial contacts between enhancers and promoters. In colorectal cancer, the distant enhancer region at FADS2, marked by rs174575, recruits the transcription factor E2F1 and facilitates physical interactions between the enhancer and the promoter [112].

Copy-number amplification in non-coding regions leads to enhancer expansion or duplication, resulting in the amplification of enhancer activity (Fig. 2B). This phenomenon drives aberrant gene expression and tumorigenesis across various cancer types. For instance, in T cell acute lymphoblastic leukemia (T-ALL), the duplication of the 8q24 region leads to the amplification of a long-range acting enhancer controlled by notch receptor 1 (NOTCH1). This amplification promotes the expression of the *MYC* gene, thereby driving T cell development and the occurrence and progression of T-ALL [113]. Based on integrative deep whole-genome analysis, a study found that most patients with metastatic castration-resistant prostate cancer have an intergenic enhancer region amplification 624 kb upstream of AR, which correlates with increased AR expression. These hotspots also appear near MYC and are associated with long non-coding RNAs that regulate post-translational modifications of MYC [114].

Viral infections are common pathogenic factors for certain cancer types, such as liver cancer, cervical cancer, and nasopharyngeal cancer (Fig. 2D) [115]. Viral insertions activate nearby oncogenes and alter gene expression networks by introducing new regulatory sequences, thereby promoting the development and progression of cancer [116, 117]. The integration of HPV generates SEs present in HPV-human hybrid ecDNA, leading to intra-chromosomal and inter-chromosomal regulation. This process results in transcription dysregulation and oncogene expression [117]. In hepatocellular carcinoma, the insertion of hepatitis B virus enhancers into the host genome activates adjacent genes, such as *TERT*, *KMT2B*, and cyclin E1 (*CCNE1*). This provides a proliferative advantage to the infected cells and promotes their malignant transformation [118]. Unlike other oncogenic viruses, the EBV genome typically does not integrate into the host genome; it exists as independently replicating episomes within infected cells. Therefore, EBV promotes enhancer “creation” in a distinct manner termed “enhancer infestation”. These episomes “forcibly” convert H3K9me3-marked heterochromatin into active enhancers marked by H3K27ac and H3K4me1 in a pioneer-like manner. This conversion further activates oncogenes and promotes tumorigenesis, such as nasopharyngeal cancer and gastric cancer [119, 120].

#### Enhancer modulation

Enhancer activity depends on the binding of TFs and specific histone modifications. Mutations occurring in TFs or epigenetic modification enzymes can reset enhancer modifications, leading to abnormal gene expression (Fig. 2C) [121]. KMT2C and KMT2D are histone methyltransferases responsible for the monomethylation of H3K4 and are frequently mutated in cancer, such as breast cancer. They regulate estrogen receptor alpha-driven (ERα-driven) transcription by activating gene enhancers. Additionally, the loss of KMT2C and KMT2D is closely associated with genomic instability and a high mutation burden in tumors and can induce epithelial–mesenchymal transition (EMT) and metastasis, thereby promoting tumor aggressiveness [122]. The p300/CBP is a lysine acetyltransferase that regulates enhancer function through histone acetylation. Mutations in p300/CBP have been identified in various solid tumors and hematologic malignancies. These mutations often result in abnormal H3K27ac at specific enhancer regions, which are associated with cancer phenotypic markers, such as proliferation, invasiveness, and metastasis [123].

The ability of mutant TFs to alter cellular functions stems from their capacity to remodel enhancers by relocating their binding sites, modifying chromatin structure, or forming new protein complexes. For instance, the nucleoporin 98-HOXA9 (NUP98-HOXA9) fusion protein in leukemia cells drives gene expression reprogramming by forming new SE [124]. Mutant p53 directly interacts with KMT2C to regulate enhancer activity and promote oncogene expression [125]. Additionally, under the tumor necrosis factor-alpha-induced (TNF-α-induced) chronic inflammatory environment, the mutant p53 reshapes the enhancer landscape by interacting with nuclear factor-κB (NF-κB), thereby promoting cancer progression [126].

### Epigenetic mechanism of enhancer reprogramming

**Fig. 3:**
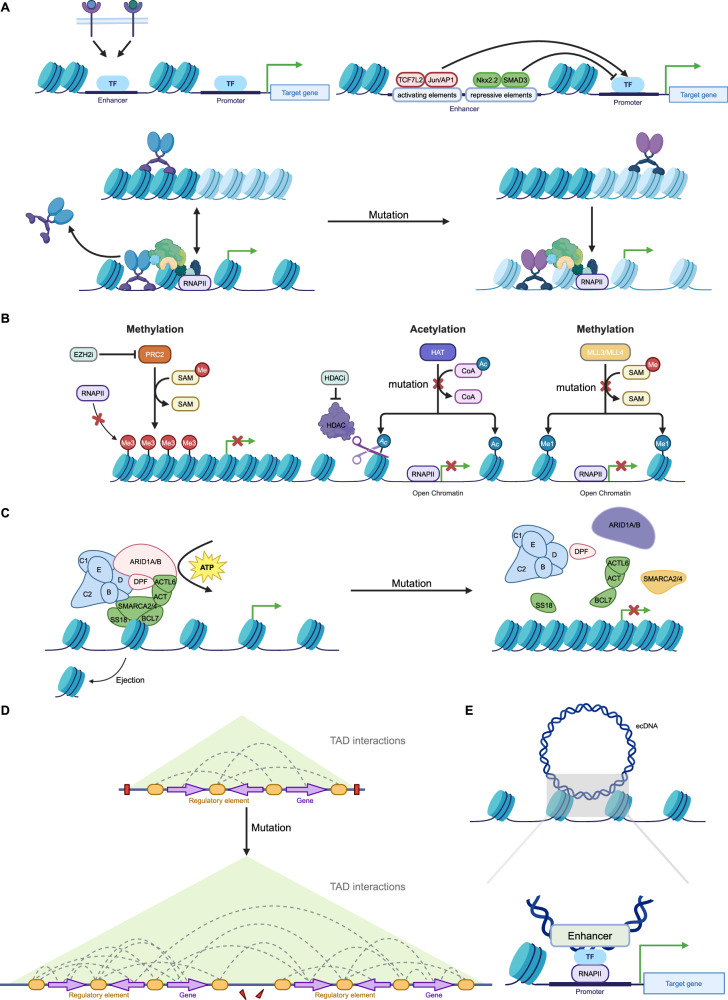
Epigenetic mechanisms of enhancer reprogramming. **A** Terminal TFs from multiple signaling pathways bind to specific enhancers to respond to environmental signals. Enhancers contain activating or repressive elements, which are controlled by different TFs. However, in cancer, repressive elements are often silenced, leading to the hyperactivity of activating elements. Mutations in pioneer factors confer non-canonical functions, reshaping enhancer activity and driving cancer progression, therapeutic resistance, and phenotypic transitions. **B** Histone modifications, regulated by “writer” enzymes such as MLL3, MLL4, CBP, and EZH2, play a crucial role in enhancer activity and gene expression. In cancer, mutations or dysfunctions in these enzymes lead to enhancer reprogramming, promoting oncogene activation and tumor suppressor gene silencing. MLL3/MLL4 mutations impair H3K4me1 deposition, disrupting enhancer function and increasing cancer cell invasiveness. CBP/p300 mutations reduce H3K27ac, suppressing immune-related genes and enhancing oncogenic transcription. Overactive EZH2 catalyzes H3K27me3 deposition, repressing key enhancers and contributing to immune evasion and differentiation blockage. **C** ATP-dependent chromatin remodeling complexes, such as SWI/SNF, regulate chromatin accessibility by modifying nucleosome positioning, with mutations in their core subunits (e.g., ARID1A, SMARCA4, SMARCA2) frequently observed in cancers. These mutations compromise chromatin remodeling, altering enhancer activity and gene expression. **D** Disruption and rearrangement of TAD boundaries in cancer enable enhancers to form abnormal interactions with oncogenes, driving their overexpression and promoting tumor progression. **E** Enhancer fragments carried by ecDNA interact with target gene promoters across the genome, leading to transcriptional reprogramming. (Created with BioRender.com).

Recent evidence suggests that epigenetic regulation might control enhancer reprogramming. Indeed, the activation process of enhancers entails intricate interactions among numerous proteins, whereby any alteration in those elements can prompt enhancer reprogramming (Fig. 3) [127, 128].

#### TFs and enhancer reprogramming

The high-order assemblies of TFs on enhancers allow precise regulation of target genes. This process is essential for regulating gene expression patterns during development, differentiation, and in response to environmental cues [129]. Previous research has revealed that terminal TFs associated with developmental signaling pathways, including the WNT, transforming growth factor beta (TGF-β), and leukemia inhibitory factor (LIF) pathways, demonstrate a predilection for binding to distinct enhancers, with a notable propensity for localizing to SEs (Fig. 3A) [130]. This evidence further supports the important role of TFs in manipulating enhancer function.

Recent investigations have elucidated that enhancers are multifaceted regulatory elements, offering both activating and inhibitory platforms for the interaction of diverse TFs [131]. The TF associated with the WNT signaling pathway, TCF7L2, along with binding motifs for the JUN/AP-1 and FOSL2 families, exhibit a significant enrichment in activating elements. Conversely, NK2 homeobox 2 (NKX2-2) and growth factor independent 1 (GFI1), both recognized for their cancer-suppressive roles, are preferentially associated with inhibitory elements (Fig. 3A) [132]. This dichotomy underscores the nuanced role of enhancers in dictating the intricate balance of transcriptional regulation, further implying their involvement in oncogenic processes.

MYC is overexpressed in most types of cancer and promotes oncogene transcription by binding to active promoters [133]. A recent study has shown that MYC binds to promoters, invades distal enhancers, and co-occupies them with cancer-type-specific TFs, such as ER and STAT3. MYC mediates the enhancer-specific recruitment of BRD4 through H3K9 demethylation and acetylation, thereby promoting the recruitment of RNA polymerase II and the transcription of eRNAs [134]. ER and AR are highly expressed in most types of breast and prostate cancer, driving cancer cell growth in response to hormones [135, 136]. Current results indicate that, upon hormone binding, ER and AR associate with distal enhancers and recruit numerous co-regulators and TFs to drive the expression of target genes [137]. Besides, TF mutations alter their binding affinity to enhancers and confer non-canonical functions, leading to enhancer reprogramming. For instance, mutant p53 (mutp53) reshapes the enhancer landscape in cancer cells by cooperating with NF-κB, significantly promoting the expression of tumor-associated genes and enhancing cell invasiveness [126]. Further research has revealed that mutp53 interacts with MLL4 to regulate H3K4 monomethylation at enhancers, thereby driving aberrant enhancer-mediated gene transcription [125]. Additionally, BRD4 has been shown to cooperate with mutp53 and eRNAs, facilitating chromatin accessibility and enhancer activation, which markedly upregulates inflammation-associated gene expression [138]. Another notable example is the recurrent mutation of CEBPA, which severely impairs enhancer activity in AML cells, suppressing immune-related gene expression. However, this dysfunction can be partially restored through LSD1 inhibition [139]. Further studies revealed that CEBPA mutations enhance its ability to bind and regulate the GATA2 enhancer, working in conjunction with TET2 mutations to rebalance GATA2 expression levels, thereby conferring greater competitiveness and aggressiveness to leukemia cells [15].

Moreover, the ability of pioneer factors to remodel the epigenome is also a vulnerability, as abnormal activity of pioneer factors has been detected in various types of cancer (Fig. 3A) [140, 141]. The FOXA family, known as archetypal pioneer factors, exhibits aberrant activity in various cancers [142, 143]. In prostate cancer, for instance, FOXA1 functions as a cofactor for the androgen receptor (AR), co-localizing at enhancers to coordinate hormone-regulated networks and promote cancer cell proliferation proliferation [144]. Androgen deprivation therapy (ADT) induces a redistribution of FOXA1 at enhancers, contributing to therapeutic resistance [137]. Moreover, FOXA1 mutations result in the acquisition of many non-canonical functions. In prostate cancer, FOXA1 mutations are generally categorized into three classes: Class-1 (missense mutations in the Wing2 region), Class-2 (C-terminal truncating mutations), and Class-3 (structural variants), which are predominantly associated with early-stage cancer, metastatic cancer, and advanced malignancies, respectively. In ER-positive breast cancer, mutations primarily occur in the Wing2 region and at the SY242CS site, corresponding to ER-dependent and ER-independent proliferative pathways. These mutations enable FOXA1 to bind novel, non-canonical DNA motifs, activating alternative gene expression programs and creating unique chromatin accessibility states. Additionally, mutant FOXA1 exhibits increased stability and activity at chromatin binding sites, driving stronger oncogene expression (Fig. 3A) [145–147]. Furthermore, in metastatic pancreatic ductal adenocarcinoma (PDA), FOXA1 becomes enriched at novel enhancer sites, driving a developmental reprogramming of PDA cells characterized by a gene expression profile resembling that of the embryonic foregut endoderm [148].

The SOX family represents another class of pioneers that bind enhancers and drive transcriptional reprogramming across various cancer types [149]. Recent studies have shown that SOX9 cooperates with TCF7L2 in gallbladder cancer to regulate each other and reprogram super-enhancers, activating multiple oncogenic signaling pathways, including ErbB and Wnt [150]. Additionally, different activation states of SOX9 during epidermal stem cell differentiation can lead to phenotypic transitions. Prolonged activation of SOX9 selectively engages tumor-associated enhancers, promoting the malignant transformation of normal cells [76]. In esophageal squamous cell carcinoma, SOX2 acquires novel genomic binding sites and collaborates with KLF5 to reprogram the epigenome. This reprogramming activates oncogenes and retroviral elements, establishing cancer cell dependency on ADAR1 [151].

While numerous studies have demonstrated that transcription factor alterations can reshape enhancers and drive phenotypic transitions in cancer cells [152], the precise mechanisms underlying these changes remain elusive. It is well established that TFs can act as both activators and repressors of transcription, but whether this dual functionality is determined by the outcome of the enhancer or the intrinsic properties of the transcription factor itself remains a topic of debate. Although some research suggests that TFs can bind different sequences within enhancers to trigger either activation or repression, the mechanisms governing this selective binding and regulation are still unclear [132]. Increasing evidence indicates that TFs often act as adaptors rather than direct regulators, exerting their effects primarily by recruiting cofactors. These cofactors influence histone modifications, chromatin remodeling, and even the 3D conformation of chromatin, thereby modulating enhancer activity and the expression patterns of target genes [153, 154]. This raises the critical question of how TFs “selectively” recruit specific cofactors to activate or repress enhancers. In fact, multiple cofactors with both activating and repressive functions coexist within cells, and their recruitment may be influenced by factors such as cell type, chromatin state, and genomic topology [155]. Some studies suggest that this selectivity may be linked to intracellular signaling pathways, chromatin accessibility, or the local environment of cis-regulatory elements [26, 156–159]. Additionally, the arrangement of TFBS, spatial conformation of enhancers, and availability of cofactors are likely to work in concert to determine the functional role of a given TF under specific conditions. Ultimately, these complex interactions construct cell-type-specific transcriptional regulatory networks, providing the molecular foundation for gene expression and phenotypic changes in cancer cells.

#### Chromatin modifications and enhancer reprogramming

Chromatin modifications primarily encompass DNA methylation and various histone modifications, such as acetylation, methylation, and phosphorylation, which regulate gene expression and influence cellular functions. DNA methylation typically occurs at CpG sites and represses gene expression by inhibiting transcription factor binding or altering chromatin structure. In contrast, histone modifications are dynamic, with different chemical modifications modulating chromatin compaction or relaxation, thereby affecting transcriptional activity. In cancer, the regulatory mechanisms of chromatin modifications are often disrupted, leading to widespread gene expression dysregulation and contributing to tumor development and progression (Fig. 3B) [160–162].

Although most enhancers are not regulated by 5mC, there is a subset of cell-type-specific enhancers that are influenced by DNA methylation. Therefore, its involvement in enhancer regions manifests in a more nuanced manner [163]. Consistent with this point, in AML, hypermethylation is associated with the silencing of enhancers, leading to the suppression of target gene expression. Although DNA hypomethylation alone is not sufficient to activate enhancers, some hypomethylated sites are associated with enhancer activation, as indicated by increased levels of H3K27ac [164]. In liver cancer, aberrant DNA methylation leads to the switching of tissue-specific enhancers during the progression of cancer, altering cell identity and affecting tumor immune surveillance [165]. The dysregulation of enhancer methylation in AML and myelodysplastic syndrome primarily manifests as hypermethylation of enhancers during myeloid lineage commitment. Mutations in tet methylcytosine dioxygenase 2 (TET2) and DNA methyltransferase 3 alpha (DNMT3A) in AML and myelodysplastic syndrome lead to either hypermethylation or hypomethylation of enhancers, thereby affecting the accessibility of specific TFBS. This epigenetic dysregulation may promote myeloid differentiation block and the development of leukemia [166].

Histone modifications constitute a highly intricate “language” of chromatin regulation [161]. In cancer, dysregulation of histone modifications is primarily driven by abnormal expression or functional alterations of epigenetic modifiers and reprogramming of metabolic pathways [98, 167]. These changes can modify the acetylation, methylation, lactylation, and other states of histones, affecting chromatin structure and gene expression. Consequently, they promote oncogene activation or tumor suppressor gene silencing, accelerating cancer progression.

MLL3 and MLL4, key “writer” enzymes responsible for depositing H3K4me1 marks at enhancers, are frequently mutated in various cancer types [168]. In breast cancer, loss of MLL3/MLL4 reduces H3K4me1 modifications at ERα target gene enhancers, impairing ERα binding and activation of these genes. This results in dysregulated expression of genes essential for cell proliferation and differentiation, increasing cancer cell invasiveness and growth [169]. Furthermore, BAP1 enhances MLL3 localization at enhancers by removing H2A ubiquitination. MLL3 mutations disrupt its interaction with BAP1, impairing enhancer localization, reducing H3K4me1 modifications, and leading to silencing of tumor suppressor genes (Fig. 3B) [170].

CBP (CREB-binding protein) and p300, histone acetyltransferases (HATs), catalyze H3K27 acetylation at enhancers to activate gene transcription [171]. Mutations in CBP/p300 represent a prominent example of enhancer reprogramming in B-cell lymphomas. These mutations lead to significant reductions in H3K27ac, resulting in sustained suppression of key enhancer functions [172–174]. For instance, in CBP-inactivated lymphomas, the BCL6-HDAC3 complex persistently represses immune-related genes such as MHC-II, leading to enhancer inactivation and enabling tumor cells to evade immune recognition [172]. In germinal center (GC) B cells, the inactivation of super-enhancers disrupts signaling pathways such as CD40 and BCR, impeding normal B-cell differentiation and promoting tumor transformation. Similarly, in follicular lymphoma, CBP mutations silence target genes of transcription factors such as FOXO1 and MEF2B, exacerbating enhancer inactivation and tumorigenesis [173]. Collectively, studies of cancer genomes indicate that CBP/p300 dysfunction constitutes a common enhancer reprogramming mechanism. By reducing H3K27ac levels, these mutations suppress tumor suppressor gene expression while enhancing oncogene transcription, leading to aberrant activation of genes involved in cell proliferation, survival, and immune evasion, thereby promoting tumor progression (Fig. 3B) [175].

H3K27me3, a hallmark repressive histone modification, is catalyzed by the PRC2 complex [176]. Alterations in EZH2, the catalytic subunit of PRC2, are strongly associated with various cancers [177]. In small-cell lung cancer (SCLC), overexpressed EZH2 represses CCL2 expression by depositing H3K27me3 at its enhancer, reducing macrophage infiltration into tumors and highlighting EZH2’s role in immune evasion. This epigenetic repression can be reversed by EZH2 inhibitors [178]. In breast cancer, EZH2 maintains luminal progenitor cells and restricts their differentiation by repressing enhancers of GATA3, a key TF driving luminal cell differentiation. EZH2 inhibitors remove this repressive mark, opening GATA3 enhancers, upregulating GATA3 expression, and synergizing with AKT inhibitors to induce differentiation and apoptosis in triple-negative breast cancer (TNBC) cells (Fig. 3B) [179].

ATP-dependent chromatin remodeling complexes, such as SWI/SNF, INO80, SWR1, and Mi2/chromodomain helicase DNA-binding protein (Mi2/CHD), harness the energy generated from ATP hydrolysis to induce DNA sliding over nucleosomes and histone exchange, thereby regulating chromatin accessibility [180]. Current research primarily focuses on the SWI/SNF complex and its core subunits, such as ARID1A, SMARCA4, and SMARCA2. Mutations in SWI/SNF components occur in approximately 20% of all cancer types [181], and are frequently associated with increased malignancy, poor differentiation, aggressive invasion, and therapeutic resistance [182–184]. These mutations compromise chromatin remodeling capabilities, particularly by altering chromatin accessibility and suppressing the expression of critical genes, thereby promoting tumor cell proliferation and survival. Notably, dual loss of SMARCA4 and SMARCA2 is prominently observed in rare, highly aggressive tumors, such as small-cell carcinoma of the ovary, hypercalcemic type (SCCOHT), and malignant rhabdoid tumors [185]. Mutations in ARID1A lead to the redistribution of enhancer-associated marks such as H3K27ac and H3K4me1, significantly increasing the expression of oncogenes (e.g., MYC, CCND1) and inflammation-related genes (e.g., IL6, TNFα), which accelerates tumor growth and facilitates immune evasion through tumor microenvironment remodeling [186, 187]. In neuroblastoma, ARID1A loss drives a transition from a neural phenotype to a mesenchymal phenotype, closely linked to enhancer reprogramming. This process activates metastasis-associated genes such as SLUG and TWIST, enhancing the invasive capabilities of tumor cells [188]. In mouse models, ARID1A mutations result in the silencing of tumor suppressor genes such as APC and TP53 while increasing the activity of oncogenic enhancers, thereby accelerating the initiation and progression of colorectal tumors [186]. In cancers with SMARCA4 mutations, SMARCA2 is often over-relied as a compensatory subunit to maintain chromatin accessibility and the expression of critical genes. It has been reported that the cooperative interaction between SMARCA2 and YAP/TEAD at enhancers is a key mechanism driving oncogene expression. Further inactivation of SMARCA2 disrupts the stability of YAP/TEAD binding at enhancer regions, thereby impairing downstream transcriptional regulatory networks (Fig. 3C) [189].

3D genome reprogramming reshapes chromatin topology by re-establishing enhancer-promoter contacts, leading to the formation of new gene regulatory circuits and the reorganization of chromatin compartments [190]. In pancreatic cancer, structural variations induce large-scale rearrangements of chromatin A/B compartments, TADs, and chromatin loops, which in turn affect the expression of cancer-driver genes such as CDKN2A and SMAD4 [191]. This 3D genome reprogramming plays a critical role not only in tumorigenesis but also in metastasis, where it is further intensified. Such reprogramming is associated with the upregulation of specific genes, including metastasis-associated genes like LIPC, whose expression is driven by metastasis-specific enhancer-promoter loops [190].

Disruption of TAD boundaries allows enhancers to rewire their connections to target genes, thereby promoting oncogene expression (Fig. 3D) [192]. In cancer, rearrangement of the TAD boundary near the IRS4 gene triggers enhancer hijacking, leading to its significantly increased expression [108]. Similarly, comprehensive epigenomic analyses have revealed widespread TAD fusion events in T-ALL, particularly involving MYC. Disruption of MYC’s TAD results in abnormal interactions between its promoter and distal enhancers, driving MYC overexpression [193]. Furthermore, chromatin interactions within TADs are significantly altered in cancer, especially between key enhancers and promoters. For instance, in T-ALL, increased chromatin interactions within TADs correlate with upregulated gene expression, and many T-ALL-specific enhancers are located within highly active TADs. Structural proteins such as CTCF and cohesin play critical roles in maintaining these interactions, enhancing chromatin interaction stability [193]. In summary, the reconfiguration of the three-dimensional chromatin structure allows enhancers to establish new contacts with promoters, leading to the formation of cancer-specific gene regulatory circuits. Disruption and rearrangement of TAD boundaries enable enhancers to bypass their original domain constraints and form aberrant interactions with oncogenes, thereby driving cancer progression.

#### Other factors and enhancer reprogramming

The ecDNA comprises small DNA fragments that are excised from chromosomes [194]. Recent research has identified that ecDNA carries mobile enhancers, which function as trans-acting activating elements and lead to the widespread deregulation of gene expression; thus, ecDNA correlates with cancer (Fig. 3E) [194–196]. A regulatory pattern similar to this mobile mechanism can also be mediated by TEs. TEs are repetitive genomic elements with binding sites for multiple TFs that exhibit enhancer characteristics [197]. Aberrant insertion of TEs into the genome can lead to dysregulation of gene expression. Furthermore, research indicates that the presence of tissue-specific TFs activates TEs across various cancer types [198].

Non-coding RNAs are integral to the orchestration of enhancer reprogramming, often acting as mediators that influence enhancer activity and gene expression. They can participate in the regulation of chromatin states by recruiting remodeling complexes, modulating the interactions between enhancers and promoters, and affecting the binding of TFs to DNA [199]. eRNAs are transcribed from active enhancer regions and might participate in various cancer signaling pathways by modulating their target genes [200]. Long non-coding RNAs can act as scaffolds for chromatin-modifying enzymes, directing them to specific genomic loci. In addition, they can sequester proteins away from chromatin, thereby influencing gene expression patterns [199].

## Critical roles of enhancers in cancer: unlocking phenotypic plasticity

### “TFs-oncogenes-enhancer” core transcription regulatory circuitry

The enhancer-driven core regulatory circuitry (CRC) encapsulates the synergistic interplay between a constellation of core regulatory factors (CRFs) and enhancers, orchestrating the intricate gene expression patterns that underpin cellular identity and functionality [201]. This paradigm holds paramount importance for elucidating the sophisticated genetic regulatory mechanisms governing cell differentiation, ontogeny, and pathological states. At the center of the CRC lies an ensemble of CRFs—predominantly master TFs—that possess the capacity to engage with cell-type-specific enhancers, thereby catalyzing distinct gene expression profiles essential for the establishment and perpetuation of cellular identity [202]. Notably, these CRFs often engage in reciprocal positive feedback loops, reinforcing cellular states and directing cellular destiny throughout the differentiation process [201]. Nevertheless, in the context of oncogenesis, this intricate regulatory network is misused, with some components being used to facilitate the delineation of cancer subtypes, while the majority are hijacked to unlock the phenotypic plasticity inherent to malignancies. This aberrant exploitation of the CRC enables cancer cells to dynamically acquire a repertoire of capabilities essential for tumorigenesis, including unbounded proliferation, apoptosis evasion, tissue invasion, and metastatic dissemination—hallmarks that epitomize malignant cells [203, 204]. Table 2 below provides a summary of the core regulatory circuits as delineated in prevalent tumors to date in different studies.

**Table. 2:** Summary of identified CRCs in different cancer types.

Cancer type	CRFs	Target gene	Reference
T-ALL	TAL1, GATA3, RUNX1	MYB, TRIB2	[110, 372]
Chronic lymphocytic leukemia	PAX5, ETV6, IRF2	BCL2, CXCR4, CD83	[373]
Acute myeloid leukemia with KMT2A (MLL)	MEF2D, IRF8	MYC, HOXA9, BCL2	[374]
B-cell precursor acute lymphoblastic leukemia	MEF2D-fusion, SREBF1, FOS, EGR1, BCL6	Genes related to pre-BCR signal	[375]
Multiple myeloma	IRF4, MYC, IKZF1, RUNX1, ETS, AP-1	-	[376]
Esophageal cancer	TP63, SOX2, KLF5	ALDH3A1, EGFR, BRD4	[203]
	ELF3, KLF5, GATA6, EHF	LIF, LIFR, HNF4A, PPARG	[221, 377, 378]
	MYC, JUNB, FOSL1	-	[379–382]
Lung cancer	ELF3, EHF, TGIF1	-	[383]
	E2F2, B-MYB, FOXM1	E2F2	[384]
	FOSL2, FOXA2, FOXA1, JUND, ATF3	-	[385]
	NKX2-1, SOX1	-	[386]
Squamous cancer	SREBF1, TP63, KLF5	Genes related to lipid metabolism and methionine metabolism	[219, 225]
Glioblastoma	SOX2, KLF4, EGR1, NOTCH1	NOTCH family	[387]
Medulloblastomas	WNT (LEF1, MAF, RUNX2, EMX2); SHH(OTX1BARHL2,MAFF,GLI2); Group3(OTX2, NRL, CRX); Group4(LMX1A, LHX2, EOMES)	CCND2, BCL2	[388–390]
Rhabdomyosarcoma	PAX3-FOXO1	KDM4B	[391]
	SOX8	MYOD, MYOG	[392]
Neuroblastoma	PHOX2B, HAND2, GATA3	-	[393]
	AP-1	-	
	TBX2	MYCN/FOXM1	[394]
	ASCL1, LMO1, MYCN, HAND2, ISL1, PHOX2B, GATA3, TBX2	ASCL1	[395, 396]
	SOX11	SMARCC1, SMARCA4, ARID1A, HDAC2, CBX2, KDM1A, c-MYB	[204]
Osteosarcoma	HOXB8, FOSL1, HOXA9	MYC, MAZ	[90]
Gastrointestinal Stromal Tumor	FOXF1, ETV1	KIT, ETV1	[397]
	HAND1	-	[398]
Gastric cancer	KLF5, GATA4, GATA6	EHF	[399]
	HNF4A, ELF3, GATA4, GATA6, KLF5	HNF1A and genes related to interleukin signal	[378]
Ewing sarcoma	KLF15, TCF4, NKX2-2	EWS-FLI1	[400]
Pancreatic ductal adenocarcinoma	Jun, Sox2, Sox5, Twist2	SOX2/5, Twist2, Nrf2	[401]
Breast cancer	BRD4, ERα	RET	[402]
	USF1, SOX4, MYBL2	Genes related to the cell cycle, Ephrin pathway, immune processes, and oxidative phosphorylation	[403]
	ESR1, FOXA1, FOSL2, JUND	-	[385]
Anaplastic large cell lymphoma	STAT3, BATF3, IRF4, IKZF1	MYC	[404]
Liposarcoma	FOSL2, MYC, and RUNX1	SNAI2	[405]
Colorectal cancer	KLF5	CCAT1	[406]
	ASCL2, KLF3, MAZ, RUNX1	-	[407]
Renal Cell Carcinoma	PAX8, HNF1B	Genes related to cell cycle and metabolism	[408]
Prostate cancer	AR, FOXA1, ERG, MYC	-	[317]
	TMPRSS2–ERG, HOXB13, FOXA1	NOTCH family	[302]
Embryonal tumors with multilayered rosettes	C19MC-LIN28A-MYCN	C19MC	[409]
Liver cancer	HNF4A, FOXA2, FOXA1, CEBPB	-	[385]
Dedifferentiated liposarcoma	FOSL2, MYC, RUNX1	SNAI2	[405]
Bladder cancer	Luminal (FOXA1, GATA3, ESR1) Basal (AP-1, SMAD2/3, NF-κB, STAT3)	Genes related to inflammatory response	[410]

### Enhancer reprogramming drives dynamic phenotypic plasticity in cancer

**Fig. 4:**
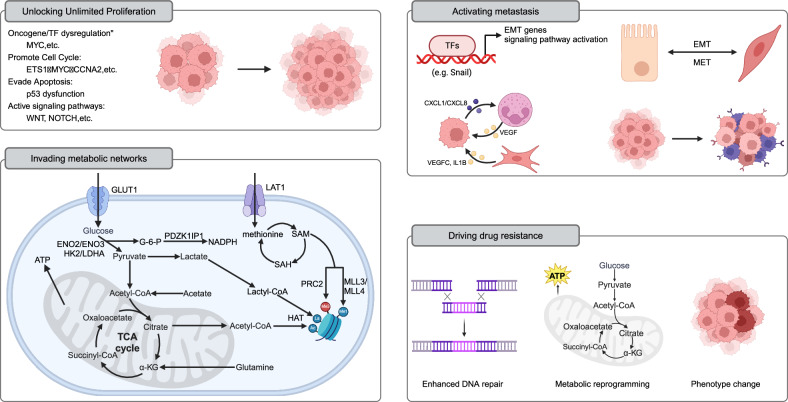
Critical roles of enhancer reprogramming in cancer. Unlocking Unlimited Proliferation: MYC, as a principal regulator of cell proliferation, undergoes enhancer reprogramming widely across various tumors, driving cancer transformation and forming a shared paradigm of foundational enhancer reprogramming in cancer. Additionally, enhancer reprogramming disrupts normal growth inhibition signals by regulating the expression of cell cycle factors (e.g., MYC, CCNA2), enabling cancer cells to sustain continuous division. It also promotes tumor growth by upregulating genes like MDM2, which suppress p53 function. Enhancer reprogramming collaborates with signaling pathways such as WNT and NOTCH to sustain infinite cancer cell proliferation by forming core regulatory circuits (CRCs). For instance, in liver cancer, WNT/β-catenin signaling activates key enhancers, driving the upregulation of the DLK1/DIO3 genomic locus and promoting tumor growth and progression. Invading metabolic networks: Enhancer reprogramming disrupts metabolic pathways in tumor cells, driving significant changes in energy production, biosynthesis, and gene regulation. Enhancers upregulate glucose transporters (e.g., GLUT1) and glycolytic enzymes (e.g., HK2, LDHA), promoting the Warburg effect and supporting anabolic metabolism. They also amplify lipid synthesis by regulating factors like SREBF1 and SREBF2, meeting the high demand for fatty acids and cholesterol in cancer cells. Similarly, amino acid metabolism is enhanced through CRC-regulated genes (e.g., LAT1), promoting tumor growth and survival. Enhancer-driven NAD metabolism reprogramming supports energy production and redox balance, further fueling cancer cell proliferation. Moreover, metabolites from these metabolic pathways, such as acetyl-CoA and S-adenosylmethionine, serve as substrates for histone and DNA modifications, establishing a feedback loop between metabolism and epigenetic remodeling to sustain tumor progression. Activating metastasis: Enhancer reprogramming plays a crucial role throughout various stages of tumor metastasis by driving the epithelial–mesenchymal transition (EMT), modulating the tumor microenvironment, and enabling immune evasion and colonization at distant sites. It activates key transcription factors (e.g., Snail, Twist) and signaling pathways (e.g., TGF-β, WNT/β-catenin), enhancing cancer cell migration, invasion, and adaptation to new microenvironments. During metastasis, enhancer-driven transcriptional reprogramming promotes organ-specific gene expression programs, such as FOXA1-mediated liver metastasis in pancreatic cancer. Additionally, epigenetic memory maintained by enhancers allows tumor cells to adapt and proliferate rapidly in distant organs (such as MET). Driving drug resistance: Enhancer reprogramming plays a pivotal role in cancer chemotherapy resistance through mechanisms such as upregulation of drug resistance genes, enhanced DNA repair, metabolic reprogramming, and lineage plasticity. (Created with BioRender.com).

A large number of altered enhancers have been identified in cancer [124]. These oncogenic enhancers act as relay stations within the cell, integrating internal and external signals and regulating various life activities of cancer cells. They dynamically provide cancer cells with the necessary growth advantages and new phenotypes (Fig. 4) [130, 205].

#### Unlocking unlimited proliferation

The oncogene MYC, a principal regulator of cell proliferation, is dysregulated across a broad spectrum of cancer types [133]. Recent investigations encompassing pan-cancer analyses have elucidated that the reprogramming of MYC gene enhancers is prevalent in a diverse array of human tumors. This evidence underscores the pivotal influence of “basic enhancer” reprogramming in the process of cancer transformation. Moreover, this implies that the occurrence of cancer may share a common paradigm of reprogrammed foundational enhancers [132].

Furthermore, enhancer reprogramming can precipitate alterations in the expression profiles of cell cycle regulatory factors. Such changes facilitate the dysregulation of the cell cycle, enabling cancer cells to circumvent conventional growth inhibition signals and continuously enter the cell division cycle [206]. For instance, YAP/TAZ serve as nuclear effectors orchestrating tumorigenesis via the Hippo pathway. Serving as TFs, they occupy enhancers with cofactor AP-1 and directly modulate target genes imperative for cell progression into the S phase and mitosis, such as ETS1, MYC, CCNA2, centromere protein F (CENPF) [74].

Under physiological conditions, cells respond to various growth inhibitory signals, such as p53-mediated apoptosis, to prevent uncontrolled proliferation [207]. While enhancer reprogramming does not typically induce mutations in p53, it can facilitate the expression of its antagonistic genes, such as MDM2, thereby neutralizing its function. Ubiquitin-specific peptidase 12 (USP12), a deubiquitinating enzyme, is driven by amplified SEs. The overexpression of USP12 shields MDM2 from degradation, leading to the ubiquitination and subsequent proteasomal degradation of p53 [208].

Certain signaling pathways, such as WNT, and NOTCH, play crucial roles in promoting cell proliferation [209]. Oncogenic enhancers facilitate infinite cancer cell proliferation by intercepting the terminal TFs of these pathways and forming CRCs [132]. Mutations in catenin beta 1 (CTNNB1) in cancer lead to increased stability of the β-catenin protein and sustained activation of the WNT/β-catenin signaling pathway. The sustained activation of β-catenin, in combination with the TCF4 complex, binds to the delta-like non-canonical Notch ligand 1- Wnt responsive element (DLK1-WRE) enhancer site upstream of maternally expressed 3 (MEG3), promoting chromatin opening and the deposition of H3K4me1 and H3K27ac. This drives the upregulation of the DLK1/iodothyronine deiodinase 3 (DLK1/DIO3) genomic locus, thus promoting the growth and progression of liver tumors [210].

#### Invading metabolic networks

Recent studies have shown that enhancer reprogramming disrupts metabolic pathways, leading to significant changes in intracellular metabolite levels. These changes alter energy production and material metabolism in tumor cells and regulate gene expression by affecting epigenetic modifications [211]. For example, the enhancer-driven MYC is closely related to glucose metabolism, lipid synthesis, and nucleotide synthesis [212].

In tumor cells, glucose is an important energy source and biosynthetic precursor [213]. Enhancers increase glucose uptake by upregulating the expression of glucose transporters, such as glucose transporter type 1 (GLUT1), and enhance the expression of glucose metabolic enzymes (such as enolase 2 [ENO2] and ENO3) [214, 215]. Additionally, enhancers activate key enzymes in the glycolysis pathway (e.g., hexokinase 2 [HK2] and lactate dehydrogenase A [LDHA]), thereby promoting the Warburg effect and increasing glycolytic flux [216, 217]. More importantly, downstream metabolites of glycolysis further support the anabolic metabolism of cancer cells and participate in the biosynthesis of ribose, amino acids, and lipids, while also regulating the intracellular redox balance. For example, SE-driven PDZK1 interacting protein 1 (PDZK1IP1) enhances the reductive capacity of colorectal cancer cells through the pentose phosphate pathway [215].

Tumor cells prefer de novo lipid synthesis, increasing the synthesis of fatty acids and cholesterol, which supports the construction of cell membranes and signal transduction [218]. SE promotes lipid synthesis pathways by regulating sterol regulatory element binding transcription factor 1 (SREBF1) and SREBF2, meeting the high demand of tumor cells for lipids [215, 219]. Furthermore, in hepatocellular carcinoma, SE-driven fatty acid synthesis-related lncRNA (FASRL) binds to acetyl-CoA carboxylase 1 (ACC1; a rate-limiting enzyme in fatty acid synthesis) and inhibits its phosphorylation, thereby promoting fatty acid synthesis [220]. The CRC formed by E74 like ETS transcription factor 3 (ELF3), KLF5, and GATA6 upregulates peroxisome proliferator-activated receptor gamma (PPARG) in esophageal cancer, leading to increased synthesis of fatty acids, phospholipids, and sphingolipids [221].

Amino acid metabolism in tumors promotes the growth and survival of cancer cells by providing energy, supporting anabolic metabolism, regulating cell signaling, and influencing epigenetic modifications [222, 223]. Of note, enhancers further amplify this effect by regulating key genes in these metabolic pathways [224]. The CRC driven by tumor protein p63 (TP63), KLF5, and SREBF1 leads to the high expression of the methionine transporter L-type amino acid transporter 1 (LAT1) in squamous cell carcinoma, significantly promoting the accumulation of methionine within the cells [225].

Nicotinamide adenine dinucleotide (NAD) is an essential coenzyme. It plays a crucial role in cellular metabolism, participating in various biochemical reactions, particularly acting as an electron carrier in redox reactions [226]. In cancer cells, NAD metabolism is significantly reprogrammed to support energy production and antioxidant defense. The increased demand for NAD^+^ in cancer cells drives their dependency on NAD synthesis pathways [227]. Recent studies have revealed enhancer-driven amplification of nicotinate phosphoribosyltransferase (NAPRT) and the dependency of cancer cells on NAPRT. Additionally, enhancer reprogramming has led to nicotinamide phosphoribosyltransferase (NAMPT), another rate-limiting enzyme in the NAD synthesis pathway, being counteracted by nicotinamide riboside kinase-dependent (NMRK1-dependent) NAD synthesis [228].

More importantly, metabolites produced by the enhancer-driven rapid metabolic network, such as acetyl-CoA and S-adenosylmethionine, serve as substrates for histone modifications and DNA methylation, directly affecting the epigenetic modifications of enhancers [211, 229]. Consistent with this, our team recently reported that during liver metastasis of pancreatic cancer, enhancer reprogramming promotes glycine amidinotransferase (GATM)-mediated guanidinoacetate metabolism. This process further facilitates pancreatic cancer liver metastasis through transcriptional activation and histone modification mediated by guanidinoacetate [230]. In summary, metabolism and epigenetic remodeling form a remarkably complex network of mutual regulation, thus promoting tumor growth.

#### Evading cell death

Apoptosis is mainly mediated through two major pathways, namely the intrinsic pathway (mitochondrial pathway) and the extrinsic pathway [231]. The permeabilization of the mitochondrial outer membrane constitutes a critical event in the intrinsic pathway, leading to the release of cytochrome c into the cytoplasm and triggering a subsequent caspase cascade, which is typically regulated by the BCL2 family [232]. In cancer, enhancer-driven overexpression of BCL2 counteracts the pro-apoptotic effects of BAX, thus protecting cells from death [233]. Notably, the use of inhibitors targeting enhancers, such as JQ1, can induce growth arrest and apoptosis in cancer cells [234]. The extrinsic pathway is initiated by extracellular signals, predominantly through the activation of death receptors, such as Fas and TNF receptors. Previous studies have reported that hypermethylation of enhancers in cancer leads to the suppression of Fas expression, thereby diminishing the sensitivity to Fas-mediated extrinsic apoptosis [235]. In addition, BRD4 exerts protective effects in various types of cancer by binding key enhancers to drive the expression of apoptosis resistance genes [236].

Furthermore, the activation of survival signaling pathways, such as NF-κB, PI3K/AKT, MAPK, and JAK/STAT, can also counteract death signals to promote the survival of cancer cells [237–239]. Enhancers provide a platform for the operation of various signal transduction pathways. On the other hand, in cancer, enhancer reprogramming results in transcriptional dysregulation of various oncogenes and TFs, perpetuating the activation of these pathways. For instance, in squamous cell carcinoma, TP63 and SOX2 activate the enhancer of EGFR, further stimulating the MEK/ERK1/2 and PI3K/AKT signaling [12].

#### Activating metastasis

The metastatic process of tumor cells commences with the invasion and migration of primary foci into adjacent tissues, in which EMT plays a significant role [240]. Research indicates that enhancer reprogramming plays a crucial role in this process by modulating the gene expression patterns of cancer cells, facilitating their transition from an epithelial to a mesenchymal phenotype. This process endows cancer cells with enhanced migratory and invasive capabilities [100]. Mechanistically, enhancer reprogramming activates key signaling pathways and TFs associated with EMT (e.g., TGF-β, WNT/β-catenin, and NOTCH) [241], as well as typical TFs (e.g., Snail, Slug, Twist, and Zeb) [242]. A recent study has demonstrated that the inhibition of FOXA2 induced by TGF-β, along with the activation of TEAD2/4, facilitates the reprogramming of a set of enhancers pre-existing within TADs, further activating the EMT process and promoting cancer cell metastasis [100]. This regulation of the gene networks intimately associated with the EMT process encompasses the suppression of epithelial markers and the expression of mesenchymal markers, the enhancement of cell adhesion, migration, and invasion, as well as the modulation of the tumor microenvironment (TME) [215, 243–245], thereby further promoting tumor metastasis and dissemination. For instance, in clear cell renal cell carcinoma, the formation of SEs robustly drives the expression of various CXC chemokines, such as CXCL8 and CXCL1. This process establishes an inflammatory immune microenvironment that facilitates neutrophil-dependent lung metastasis [246]. Of note, research indicates that the enhancer-driven pro-metastatic microenvironment extends beyond cancer cells. The remodeling of enhancers within other components of the TME also significantly impacts cancer progression. For instance, the activation of JUN is underscored as a pivotal factor in the activation of cancer-associated fibroblast-specific (CAF-specific) enhancers, promoting the expression of pro-metastatic genes and, thereby, augmenting breast cancer invasiveness in a non-cancer-cell-autonomous manner [247]. This suggests that enhancer reprogramming within the stromal cells of the TME plays a significant role in facilitating cancer progression and metastasis.

Notably, enhancer reprogramming amplifies the transcriptional output of metastasis-associated genes and orchestrates a complex transcriptional network that collectively enhances the metastatic potential of cancer cells, such as those associated with embryonic or stem cell-like properties, which are closely related to the metastatic capabilities of cancer cells [148, 248]. For instance, the activation of FOXA1-dependent enhancer drives an embryonic foregut endoderm transcriptional program, rendering pancreatic cancer cells more invasive and facilitating their liver metastasis [148].

Upon their departure from the primary site, cancer cells directly encounter immune cells within the circulation. Prior research has shown that cancer cells may evade immune surveillance through interactions with blood cells, such as platelets and neutrophils [249, 250]. Nonetheless, the specific role of enhancer reprogramming in this context remains to be elucidated.

Studies have demonstrated that enhancers can elevate the expression of immune checkpoint inhibitors, such as programmed cell death-ligand 1 (PD-L1), thus promoting immune escape [251]. However, the potential exploitation of this mechanism by circulating tumor cells remains under investigation. The tumor cells that survive the circulatory journey and arrive at new locations are similarly subjected to the pressures of colonization. During this phase, enhancer reprogramming facilitates the successful settlement of tumor cells and the formation of new tumors by modulating genes associated with adaptation to the microenvironment, cellular proliferation, and evasion from immune responses [243, 252]. Additionally, cancer cells that have experienced EMT may undergo a reversible process during the colonization stage, namely the mesenchymal–epithelial transition, thereby regaining epithelial characteristics to promote growth. Intriguingly, it was recently discovered that epigenetic memory explains this phenomenon. Certain enhancers, which are induced to shut down during the EMT process, do not directly enter a quiescent state; instead, they maintain a certain level of H3K4me1 modification, remaining in a primed state. When reaching a new microenvironment, these enhancers are rapidly reactivated, driving the cell transformation into an epithelial phenotype [100]. Furthermore, metastatic tumor cells may enter a state of dormancy rather than immediately forming new, a condition that might persist for years. Nevertheless, these cells are capable of rapidly resuming proliferation in response to microenvironmental signals. Recent research suggests that this phenomenon can also be explained through epigenetic memory. For instance, a set of enhancers regulated by TFs (e.g., SOX9) and located within variable chromatin structures, can activate a strong transcriptional response upon exposure to retinoic acid [252].

Cancer cells of the same type tend to metastasize to specific organs; however, the precise underlying mechanisms remain unclear [253]. Recent evidence increasingly indicates that enhancer-driven transcriptional reprogramming plays a significant role in this process. For instance, during liver metastasis of pancreatic cancer, the original pancreatic developmental program is replaced by a liver developmental program, achieved through FOXA1-driven enhancer reprogramming [148]. Similarly, in colorectal cancer cells metastasizing to the liver, enhancers acquire liver-specific TFs, FOXA2 and HNF1A, thereby activating liver-specific gene transcription. Surprisingly, further transcriptomic analyses across various cancer types revealed that similar transcriptional reprogramming occurs in distant metastases of other cancers, such as colon cancer to the lung, prostate cancer to the bone, kidney cancer to the lung, and breast cancer to the brain [254]. RUNX2 is a crucial TF associated with bone development and exhibits pioneer activity [255]. In prostate cancer, FOXO1 binds to and inhibits RUNX2-mediated bone metastasis, a finding supported by clinical data [256]. Overall, these results strongly support the notion that organotropic metastasis of cancer may be mediated by acquired enhancer-driven transcriptional reprogramming. Changes in transcriptional programs confer metastatic cancer cells with characteristics of distant organs, aiding their adaptation and colonization. These findings provide new insights into the mechanisms of cancer metastasis and lay the theoretical foundation for developing novel therapies against metastasis.

In summary, the above results underscore the regulatory role of enhancer reprogramming throughout various stages of tumor metastasis. Nonetheless, tumor metastasis remains an intricately coordinated process, involving numerous mechanisms that remain to be elucidated. For instance, extensive research has highlighted the role of exosomes in metastasis and their significance in establishing pre-metastatic niches in distant organs [257]. Concurrently, eRNAs have been identified within exosomes and are associated with the long-range regulatory functions of enhancers [258]. However, the mechanism through which enhancers regulate pre-metastatic niches has not been thoroughly investigated. Following the recent discovery of epigenetic memory, the mechanism by which tumor cells remember and respond to changes in their microenvironment at the molecular level has become clearer. This mechanism elucidates the process of tumor cell dormancy and resuscitation and reveals the process through which tumors rapidly adapt to new environments and promote proliferation upon distant colonization. Epigenetic memory, by maintaining the active state of specific enhancers, enables tumor cells to swiftly activate or suppress the expression of particular genes at opportune moments, thus playing a pivotal role during metastasis and colonization [32]. These insights provide crucial clues for the development of new therapeutic strategies targeting tumor metastasis, especially those aimed at disrupting the epigenetic memory of tumor cells to block their metastatic and colonization capabilities.

#### Driving drug resistance

Studies have proposed several mechanisms of chemotherapy resistance in cancer that are mediated by enhancer reprogramming, including the upregulation of drug resistance genes, enhanced cell proliferation [206], resistance to cell death [259], enhanced DNA damage repair [260], reactivation of signaling pathways [261], stemness [262], metabolic reprogramming [263], and lineage plasticity [155, 264].

Based on the concept of synthetic lethality, poly (ADP-ribose) polymerase (PARP) inhibitors (PARPi) have been extensively utilized in the treatment of tumors carrying BRCA mutations (e.g., breast and pancreatic cancers) to target cancer cells with homologous recombination repair (HRR) deficiencies [265]. However, resistance to PARPi partially limits their clinical application, with the principal mechanism of resistance being the restoration of HRR [266]. A recent study in ovarian cancer has demonstrated the presence of SEs at the KLF5 locus and formed CRC. Elevated expression of KLF5 promotes the expression of key HRR pathway genes, including RAD51, checkpoint kinase 1 (CHEK1), RAD54 like (RAD54L), essential meiotic structure-specific endonuclease 1 (EME1), and Bloom syndrome (BLM), thereby reversing the HRR suppression mediated by PARPi. Moreover, epigenetic targeting of KLF5 can restore sensitivity to PARPi [260].

Dysfunction in signaling pathways emerges as a critical mechanism underlying acquired drug resistance. Targeted therapeutic approaches designed to inhibit specific signaling pathways are significantly challenged by the reactivation of these pathways, which leads to drug resistance [267]. Serving as hubs for multiple signaling pathways, enhancers contribute to tumor growth recovery by reactivating targeted signaling pathways or activating alternative bypass routes. For instance, the downregulation of MAPK pathway negative regulators, mediated by enhancer reprogramming, leads to the reactivation of the MAPK pathway, resulting in resistance to MEK inhibitors. However, the combination of histone deacetylase (HDAC) inhibitors (i.e., HDACi), can effectively overcome this resistance [261].

Prolonged use of fibroblast growth factor receptor (FGFR) inhibitors may lead to the suppression of SWI/SNF, thereby activating YAP-dependent enhancers. This, in turn, alters the mechanistic target of the rapamycin kinase-mediated (mTOR-mediated) amino acid metabolic pathway, leading to tumor growth [263]. Additionally, under BRAF/MEK inhibitor treatment, enhancers regulated the formation of new transcriptional states and activated oxidative phosphorylation as the primary energy source for drug-resistant myeloma cells [268]. The above evidence supports the connection between metabolic reprogramming and cancer drug resistance, highlighting the role of enhancers in this process.

Lineage plasticity refers to the evolution of cells from one differentiated state to another. Recent studies have discovered that such transformations can determine therapeutic responses [269]. Therapy-induced shifts in enhancer landscapes facilitate phenotypic transitions in cancer cells, leading toward drug resistance [270]. During the treatment of lung adenocarcinoma with KRAS inhibitors, a subset of patients exhibits adeno-to-squamous transition, which is associated with the activation of the enhancer for the squamous lineage TF ΔNp63. The upregulation of ΔNp63 activates signals for the adeno-to-squamous transition plasticity signature, thereby promoting KRAS inhibitor resistance [264]. In breast cancer, the ERα, along with other oncogenic TFs (e.g., GATA3 and AP-1), drives global enhancer reprogramming and the reconfiguration of transcriptional networks. This leads to the transdifferentiation of breast cancer cells from a luminal/epithelial state to a basal/mesenchymal phenotype, thereby acquiring endocrine resistance [155]. Similarly, in prostate cancer, the inhibition of the AR pathway activates changes in the FOXA2-dependent transcriptional pattern, driving the adeno-to-neuroendocrine transition, thereby causing castration resistance [271].

Taken together, the multifaceted role of enhancer reprogramming in cancer resistance not only enhances our understanding of complex resistance mechanisms but also provides a foundation for developing new treatment strategies. These strategies may include direct interventions targeting enhancer activity, or reversing drug resistance by altering epigenetic status, providing new directions for cancer treatment.

#### Maintaining stemness

CSCs are typically characterized by functions such as sustaining self-renewal, initiating tumor development, and mediating drug resistance. Recent reviews have delved into the epigenetic regulatory mechanisms involved in the formation of CSCs [272].

Typically, enhancers maintain stemness by driving the expression of stem cell TFs, such as SOX2, OCT4, c-MYC, and KLF4 [203, 273]. These TFs are pivotal in sustaining the core regulatory network essential for stem cell identity, self-renewal capabilities, and pluripotency. Enhancer reprogramming further modulates the chromatin landscape, rendering it more permissive for the transcriptional machinery to access key stemness genes, such as TP63, MET, FOSL1, and X-box binding protein 1 (XBP1) [248, 274, 275]. This ensures the continuous expression of genes that are critical for maintaining a stem-like state and allows for the rapid adaptation of cancer cells to environmental stresses or therapeutic interventions. Additionally, this reprogramming supports the phenotypic plasticity of cancer cells, enabling transitions between different cellular states, for instance, in breast cancer, overexpression of MYC triggers phenotypic transitions and induces a stem cell-like state [276]. Therefore, the role of enhancers extends beyond the mere activation of individual genes; it encompasses the orchestration of complex gene networks that collectively uphold the stem cell-like characteristics within tumors, presenting a significant challenge and opportunity for cancer therapy.

### Enhancer-based subtypes of cancer

The specific tissue distribution is among the most remarkable features of enhancers. Most enhancers are inactive in a given tissue or organ, and only a few that determine cell identity are activated and involved in cell fate determination [54]. This characteristic is maintained in tumors. Genome-wide studies have shown that different cancer subtypes show significantly varied enhancer maps. In other words, different enhancer landscapes drive the emergence of different cancer subtypes [277].

Many studies have revealed the potential of enhancers in profiling cancer subtypes. Wu et al. previously summarized the relevant reports, and we made some additions on this basis [277] (Table 3).

**Table. 3:** A summary of published studies on enhancer-based subtypes of cancer.

Cancer type	Size of sample	Features for subtyping	Identified/Characterized subtypes	Multi-omics characterization	References
Acute myeloid leukemia	66 patients and 28 cell lines	SE H3K27ac signal	Six novel subtypes: C1, C2, C3, C4, C5, C6.	H3K27ac ChIP-seq; somatic mutation; RNA-seq,	[411]
Gastric cancer	23 patients and 26 cell lines	SE H3K27ac signal	Two subtypes: Mes and nMes	H3K27ac and H3K4me1 ChIP-seq; ATAC-seq	[412]
Gastric cancer (Peritoneal metastasis)	98 patients and 59 cell lines	SE H3K27ac signal	Two subtypes: EMT group (characterized by SMAD3 as the only master TF, followed by RUNX1, BHLHE40 and TEAD1) and non-EMT group (enriched in ELF3 and KLF5)	H3K27ac ChIP-seq; DNA methylation analyses; RNA-seq; WGS analyses; CNV	[413]
Pancreatic cancer	24 human PDAC samples grown as patient-derived tumor xenografts (PDTXs)	SE H3K27ac signal	Two subtypes: the classical subtype and the basal subtype	H3K4me1, H3K27ac, H3K4me3 H3K27me3 and H3K9me3 ChIP-seq; whole-genome DNA methylation analysis; RNA-seq; SNP arrays analysis	[414]
Lung cancer (lung adenocarcinoma,LUAD)	42 patients	SE H3K27ac signal	Two groups: GI represents the more aggressive tumor while GII represents the less aggressive one.	H3K27ac ChIP-seq, RNA-seq	[415]
Lung cancer (lung squamous cell carcinoma, LUSC)	13 cell lines	SE H3K27ac signal	Two subtypes: the novel “neural” subtype, defined by SOX2 and Brn2, and “classical” subtype, defined by SOX2 and p63.	H3K27ac ChIP-seq; RNA-seq	[416]
Lung cancer (small-cell lung cancer, SCLC)	25 cell lines	SE H3K27ac signal	Four known clusters: SCLC-A, SCLC-N, SCLC-P, SCLC-Y, and two novel subtypes among SCLC-A: SCLC-Aα and SCLC-Aσ.	H3K27ac ChIP-seq; RNA-seq	[386]
Neuroblastoma	60 patients and 25 cell lines	SE H3K27ac signal	*MYCN*-amplified, *MYCN* non-amplified high-risk, *MYCN* non-amplified low-risk; A novel subtype, exhibiting mesenchymal characteristics, shared cellular identity with multipotent Schwann cell precursors, and RAS activated.	H3K27ac and H3K4me3 ChIP-seq; HiCHiP; ATAC-seq; RNA-seq	[417]
Prostate cancer	100 patients	SE H3K27ac signal	Three clusters: Cl1, Cl2 and Cl3	AR, H3K27ac, H3K4me3, and H3K27me3 ChIP-seq; RNA-seq	[418]
Glioblastoma	50 fresh-frozen tumor specimens, 20 patient-derived GPCs, and 5 established GBM cell lines	SE H3K27ac signal	Four subtypes: AC1-mesenchymal, AC1-classical, AC2-proneural, and AC3-proneural	H3K27ac ChIP-seq; RNA-seq; Sanger sequencing	[419]
Medulloblastoma	28 patients and 3 cell lines	SE H3K27ac signal	Four subtypes: WNT, SHH, Group 3, and Group 4.	H3K27ac, BRD4, H3K27me3, H3K4me1, LMX1A, LHX2, and HLX ChIP-seq; RNA-seq; 4C-seq; DNA methylation, CNV	[390]
Breast cancer	19 cell lines	SE H3K27ac signal	Two groups: TNBC and non-TNBC	H3K27ac, H3K4me1, H3K4me3, H3K27me3 ChIP-seq	[420]
Colorectal cancer	69 samples and 11 cell lines	SE H3K27ac signal	Four subtyps: EPiC1,EPiC2,EPiC3 and EPiC4.	H3K4me1, H3K4me3,H3K79me2,H3K9me3 and H3K27me3 ChIP-seq	[421]
Renal cell carcinoma	42 patients	SE H3K27ac signal	Three subtypes: ccRCC, pRCC, chRCC	H3K27ac and H3K4me2 ChIP-seq; ATAC-seq; RNA-seq; SNP arrays analysis	[422]
Bladder cancer	4 patients and 4 cell lines	SE H3K27ac signal	Two subtypes: luminal and basal.	H3K27ac ChIP-seq; RNA-seq; ATAC-seq; Hi-C	[423]
	15 patients and 9 cell lines	SE H3K27ac signal	Two subtypes: luminal and basal.	H3K27ac, H3K27me3 and H3K9me3 ChIP-seq; RNA-seq	[410]
Multiple myeloma	30 patients and 5 cell lines	Chromatin accessibility and paired transcriptome profiles	Four subtypes: MAF, CCND1, HD, and MMSET.	H3K27ac ChIP-seq, ATAC-seq; RNA-seq	[424]

### Enhancer reprogramming and TME

**Fig. 5:**
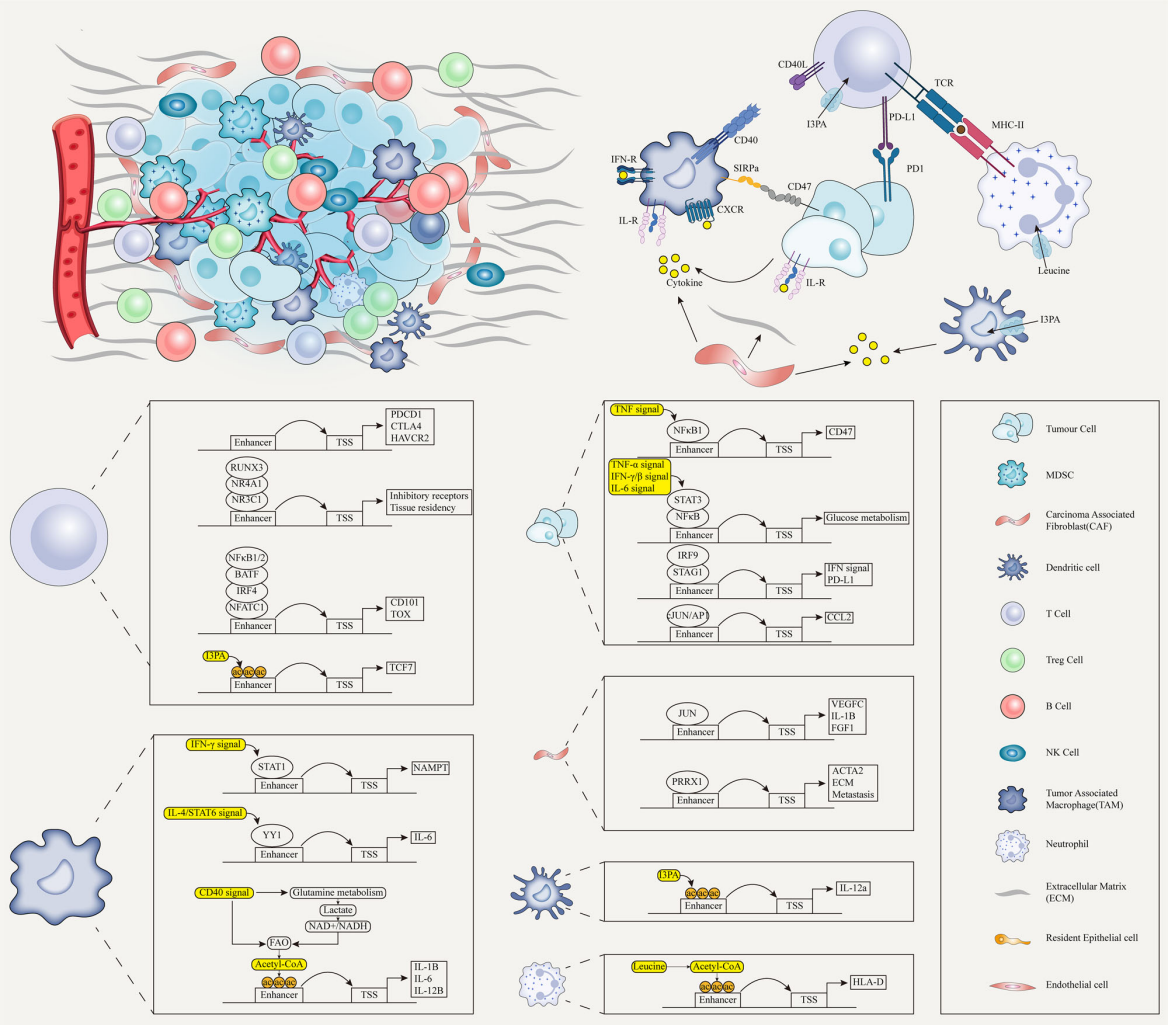
Enhancer reprogramming and TME. Different cells in the tumor microenvironment respond to various signals (such as cytokines, intercellular receptor-ligand interactions, metabolites, etc.) to regulate enhancer-driven transcriptional programs. These signals modulate enhancer activity, driving the reprogramming of cancer cell transcriptional programs and remodeling of the tumor microenvironment.

The TME is a highly complex ecosystem, consisting of a variety of cellular and non-cellular components, which profoundly influences the evolution of cancer [278]. Extensive research has unveiled the critical impact of enhancer-driven TME remodeling on tumor progression, treatment response, and resistance to therapy. Within this biological framework, enhancer reprogramming serves as a mechanism driving the intrinsic transformation of cancer cells and directly shapes the dynamic changes in the TME. This regulatory mechanism encompasses aspects ranging from immune cell modulation and extracellular matrix remodeling to angiogenesis (Fig. 5) [279–281].

Despite the substantial infiltration of T cells within the TME, a significant portion inevitably progresses toward an exhausted state (exhausted T cells [TEX]) [282]. A recent study reported on the epigenetic mechanisms underlying the heterogeneous function and dysfunction status of tumor-infiltrating lymphocytes. Specifically, six groups of TME-infiltrated CD8+ T cells were defined based on their chromatin status and connected to specific gene and enhancer/promoter pairs. Clusters 1 and 2 were associated with exhaustion, characterized by high programmed cell death 1 (PD-1) expression and activity of exhaustion markers, such as hepatitis A virus cellular receptor 2 (HAVCR2), thymocyte selection associated high mobility group box (TOX), TOX2, and layilin (LAYN). Cluster 3 was associated with progenitor TEX (TPEX), while clusters 4 and beyond represented memory, cytotoxicity-associated, and heat-shock response genes, indicating diverse functional states within the T cell population [283]. Using single-cell ATAC-seq technology to analyze tumor-infiltrating T cells, 19 subpopulations were identified. In TEX cells, enhancers associated with inhibitory receptor genes were discovered, such as the +5 kb enhancer of PDCD1 (encoding PD-1), which showed specific accessibility in TEX. Similarly, distal enhancers at the cytotoxic T-lymphocyte associated protein 4 (CTLA4) and HAVCR2 gene loci also exhibited specific activity in TEX, further supporting the notion that the exhausted state may be regulated by state-specific enhancers. Additionally, TEX undergoes two stages during differentiation. The first stage (intermediate exhaustion stage) involves increased accessibility of cis-regulatory elements near inhibitory receptor genes, while the second stage (terminal exhaustion stage) involves increased accessibility of cis-regulatory elements near genes associated with terminal T cell dysfunction, such as CD101 and TOX [280]. Notably, the exhausted state of T cells appears to be reversible. The indole-3-propionic acid (I3PA) produced by the cooperation between Lactobacillus johnsonii and Clostridium sporogenes in the gut increases the H3K27ac at the SE of TCF7 in TPEX, reactivates TPEX, and maintains stemness in CD8+ T cells [284]. Additionally, I3PA promotes the production of IL12a by increasing the deposition of H3K27ac at the IL12a enhancer in dendritic cells, thereby enhancing the function of tumor-infiltrating CD8+ T cells [285]. Furthermore, enhancer reprogramming within cancer cells can also promote the expression of PD-L1 on the cell surface, thereby mediating immune escape [251, 286, 287].

The interactions between tumor-associated macrophages (TAMs) and cancer cells, as well as stromal cells within the TME, through mechanisms of enhancer reprogramming, can further sustain and exacerbate various characteristics of cancer [288]. Local IFN-γ signaling activates STAT1 and manipulates the intronic enhancer of nicotinamide phosphoribosyl transferase (NAMPT). Subsequently, NAMPT influences the glucose metabolism and tricarboxylic acid cycle of TAMs, driving the anti-tumor reprogramming of TAMs [289]. Similarly, the activation of the CD40 signal also leads to the activation of enhancers at pro-inflammatory gene loci in TAMs through metabolic reprogramming, thereby coordinating the polarization of the anti-tumor phenotype [290]. Conversely, enhancer-driven C-C motif chemokine ligand 2 (CCL2) production in pancreatic ductal adenocarcinoma leads to the recruitment of TNF-α-positive macrophages and triggers the transition from a “classical” subtype to a more aggressive “basal-like” subtype [288]. Intriguingly, drug-induced enhancer activation can reprogram the M2-type into the M1-type, thereby restoring the anti-tumor effects of macrophages [291]. In summary, these results highlight the crucial role of enhancer reprogramming in modulating the interactions between TAMs and TME, thereby significantly impacting cancer progression.

Within the complex ecosystem of the TME, neutrophils (tumor-associated neutrophils [TANs]) emerge as pivotal players whose functions extend beyond traditional roles in inflammation and immune defense [292]. A recent report identified 10 states of TANs, demonstrating their tissue and phenotypic plasticity. Diversity in metabolic pathways may be associated with this phenomenon, further suggesting a complex interplay between metabolic reprogramming and the multifaceted roles of TANs within the TME. Intriguingly, among them, leucine metabolism facilitates the activation of the SE of major histocompatibility complex-II (MHC-II) through the production of acetyl-CoA, thereby enhancing the antigen presentation mechanism and effective activation of T cells [293]. Besides, cancer cell-derived cytokines could shape an inflammatory TME, thereby triggering neutrophil-dependent metastasis [246]. These studies suggest that the activity and behavior of TANs are intricately regulated by epigenetic mechanisms, among which enhancer reprogramming stands out as a key modulator.

Additionally, CAFs stand out as key architects in the stromal component and contribute to the creation of a protective niche for cancer cells through epigenetic modulation [294]. For example, cytokines secreted by CAFs, such as IL6 and IL8, can promote the phosphorylation of the BRD4 protein. In turn, BRD4 binds to enhancers, inducing chromatin remodeling and supporting the transcriptional mechanisms within cancer cells [295]. Within CAFs, enhancer reprogramming is one of the key mechanisms driving the transition from normal fibroblasts to promoters of cancer progression. For example, in breast cancer, studies have found that the activation of the JUN TF binding to specific enhancers in CAFs drives the reprogramming of these enhancers, thus activating the expression of a series of genes that promote tumor invasiveness and metastasis [247]. Paired related homeobox 1 (PRRX1) has been identified as a master TF controlling the myofibroblastic lineage progression in CAFs. Through integrated gene expression and chromatin accessibility analyses, studies have found that PRRX1 drives CAF activation by reprogramming enhancer activity, such as upregulating actin alpha 2, smooth muscle (ACTA2), and other genes involved in extracellular matrix remodeling and cell migration [296].

The presence of inflammatory clues is another important feature of the TME [297]. The TNF-NFκB1 signaling pathway directly regulates the expression of CD47 by binding to a constituent enhancer within a breast cancer-specific SE. In turn, CD47 inhibits the phagocytic activity of immune cells (e.g., macrophages) by interacting with the SIRPa receptor on these cells [298]. Mutations in stromal antigen 2 (STAG2) activate interferon regulatory factor 9 (IRF9) by regulating 3D genome organization, thereby enhancing type I interferon signaling and increasing PD-L1 expression. This process is associated with the replacement by STAG1, as well as increased H3K27ac signaling and the formation of new E-P loops [299]. Additionally, TNF-α, IFN-γ, and IL6 signals induce SE formation in colorectal cancer, with inflammatory signaling further directly regulating their activity through NF-κB and STAT3 [215].

Overall, these findings provide a comprehensive overview of the crucial role of enhancer reprogramming in the TME, impacting the dynamics between cellular and non-cellular components. It highlights the complexity of the TME, where cellular and epigenetic mechanisms (including enhancer reprogramming) drive significant changes in immune cell function, thereby contributing to cancer progression, immune escape, and therapy resistance.

## Targeting carcinogenic enhancers: promising strategies for transcription network normalization

Targeting carcinogenic enhancers presents a groundbreaking approach in the quest to normalize the transcription networks that are pivotal for cancer progression. Dysregulation of these regulatory elements is instrumental in activating oncogenic pathways and sustaining the malignant phenotype of cancer cells. By specifically addressing these enhancers, researchers aim to disrupt the aberrant transcriptional programs that drive tumor growth and resistance mechanisms. This strategy holds the potential to precisely curtail the expression of oncogenes and restore the normal regulatory circuits of the cell, offering a promising direction for cancer therapy that targets the genetic underpinnings of the disease. As we delve deeper into the molecular intricacies of enhancer function within cancerous cells, the opportunity to redefine therapeutic interventions becomes increasingly tangible, marking a significant shift toward more targeted and effective cancer treatments.

The therapeutic strategies targeting enhancers that have been proposed to date are based on various stages of enhancer activation, as well as the ablation of the transcription machinery [300]. Given that terminal TFs of multiple signaling pathways frequently occupy enhancers, the use of pathway inhibitors to block the transmission of external signals to enhancers is an effective strategy [132]. Using the JAK pathway inhibitor ruxolitinib to inhibit IL6/STAT3 signaling disrupts the ER-FOXA1-pSTAT3 enhancer-driven transcriptional program of target genes in breast cancer and suppresses the invasive capacity of cancer cells [301]. Similarly, NOTCH inhibitors antagonize the function of ETS-related gene-dependent (ERG-dependent) enhancers and inhibit the growth and invasiveness of prostate cancer cells [302].

Another important approach is targeting the epigenetic modifications of enhancers. Some drugs disrupt enhancer histone modifications by inhibiting DNMTs, HDACs, and HATs [161]. Several small molecule inhibitors of CBP/p300 have been developed [303]. A-485 binds to the catalytic active sites of p300 and CBP, competitively inhibiting the binding of acetyl-CoA, thereby significantly impairing acetylation deposition. A-485 demonstrates lineage-specific antiproliferative activity in 124 cancer cell lines, particularly showing significant effects in hematologic malignancies and AR+ prostate cancer [304]. Similarly, inobrodib (CCS1477) reduces the gene expression driven by AR and C-MYC in prostate cancer [305]. Additionally, a novel chemical degrader of CBP/p300, namely dCBP-1, eliminates key enhancers driving MYC expression and significantly inhibits the growth of multiple myeloma cells [306]. Compared with CBP/p300, several HDACi have been approved for the treatment of cancer [307]. Recent studies have also examined novel HDACi and combined therapy, demonstrating improved efficacy in cancer therapy [308–311]. Targeting DNMT can remove abnormal DNA hypermethylation and, consequently, rescue the genes that have been silenced. In cancer cells that enter a dormant state in response to TGF-β, the stimulator of interferon genes (STING) promoter and enhancer exhibit high methylation and chromatin suppression, leading to reduced STING activity. Using DNMT inhibitors can rescue STING expression, triggering the expression of interferons and pro-inflammatory chemokines, thereby enhancing immune recognition and clearance [312]. Enhancer of zeste 2 polycomb repressive complex 2 subunit (EZH2) is the catalytic subunit of the polycomb repressive complex 2 (PRC2) and is responsible for H3K27me3. EZH2 is overexpressed or dysfunctional in numerous types of cancer, and EZH2 inhibitors exert their anti-tumor effects by blocking its methyltransferase activity, thereby reactivating silenced genes [176]. IHMT-337 covalently binds to EZH2 and degrades it through a ubiquitin-dependent pathway, thereby inhibiting the proliferation of breast cancer and diffuse large B-cell lymphoma [313]. Currently, several EZH2 inhibitors, such as valemetostat tosilate (Ezharmia) and tazemetostat (Tazverik), have been approved for the treatment of cancer [314, 315].

In cancer therapy, targeting the SWI/SNF complex shows great potential, as disrupting its key subunits can significantly impact chromatin structure and gene expression [316]. AU-15330 is a highly selective degrader targeting SMARCA2 and SMARCA4, which induces a specific loss of chromatin accessibility at enhancers in prostate cancer cells driven by AR and FOXA1. This disruption affects the core enhancer circuits that are dependent on these TFs, thereby eliminating the downstream oncogenic gene programs [317]. During SWI/SNF ablation, enhancers remain persistently repressed; however, the accessibility and transcriptional activity of many promoters are restored, possibly due to the compensatory action of the EP400/TIP60 coactivator complex. Simultaneous inhibition of EP400/TIP60 can enhance the sensitivity to SWI/SNF inhibition [318].

Although the effects of TFs on enhancers are well established, directly targeting TFs remains a challenge [319]. However, TFs participate in the assembly of the transcription machinery by recruiting cofactors, such as CDKs and BRD4. These approaches have been thoroughly summarized in previous articles, hence will not be elaborated further here [320–322]. Instead, building on this foundation, we aim to explore innovative avenues that extend beyond traditional interventions.

Combination therapy can achieve higher efficiency for tumor cell clearance in a shorter period of time [323]. For example, cotreatment with bromodomain antagonist, HDACi, and CDK4/6 inhibitors can synergistically induce apoptosis in ibrutinib-resistant mantle cell lymphoma cells [324]. The combined use of HDACi overcomes the resistance to MEK inhibitors caused by the downregulation of MAPK pathway negative regulators [261]. More importantly, increasing evidence suggests that enhancer perturbation can activate immune responses, potentially involving interferon signaling pathways, tumor immunogenicity, and pyroptosis [281].

Therefore, combined immunotherapy is a promising treatment strategy(Table 4). A recent study combining PD-1 monoclonal antibodies, HDACi, and vascular endothelial growth factor (VEGF) monoclonal antibodies showed improved prognosis in colorectal cancer [308]. A phase II clinical study combining anti-PD-1 and HDACi in peripheral T cell lymphoma is also currently underway [325].

**Table. 4:** Summary of clinical trials combining epigenetic inhibitors with other treatment modalities.

Drug	Disease	NCT Number	Interventions	Phase
HDACi	Relapsed/refractory classical Hodgkin lymphoma	NCT06393361	1. Chidamide; Decitabine; Anti-PD-1 Antibody 2. Brentuximab Vedotin, anti-PD-1 antibody.	II
		NCT06563778	1. Chidamide; Decitabine; Anti-PD-1 Antibody 2. Brentuximab Vedotin+ Bendamustine+Anti-PD-1 antibody	II
	Hodgkin Lymphoma	NCT04514081	1. Chidamide; Decitabine; Camrelizumab 2. Decitabine+Camrelizumab	II
		NCT04233294	Chidamide; Camrelizumab; Decitabine	II
	Non-Hodgkin Lymphoma	NCT04337606	Chidamide; Decitabine; Camrelizumab	I/II
	1. Relapsed/Refractory Non-Hodgkin Lymphoma 2. Advanced Solid Tumors	NCT05320640	Chidamide; Decitabine; Immune checkpoint inhibitors (anti-PD-1/PD-L1/CTLA4 antibodies)	I/II
	Neuroendocrine Tumors	NCT05113355	Chidamide; Sintilimab	II
	Multiple Advanced Cancers	NCT04708470	Bintrafusp Alfa; PDS01ADC; Entinostat	I/II
EZH2i	Multiple Advanced Cancers	NCT06022757	XNW5004; KEYTRUDA® (pembrolizumab) 25 mg/mL Solution for Injection	Ib/II
	HR+/HER2- endocrine-resistant advanced breast cancer	NCT04355858	SHR3162; SHR2554	II
	1. Advanced Solid Tumor 2. Lymphoma	NCT04407741	1. SHR2554 and SHR1701 2. SHR1701	I/II
	Follicular Lymphoma	NCT05551936	Bendamustine; Rituximab; Tazemetostat	I/II
	Recurrent Ovarian Cancer	NCT05942300	CPI-0209; Carboplatin	I
	Metastatic Castration-Resistant Prostate Cancer	NCT06629779	PF-06821497; Placebo; Enzalutamide	III
		NCT06551324	PF-06821497; Docetaxel; Enzalutamide	III
	Follicular Lymphoma	NCT04224493	Tazemetostat; Lenalidomide; Rituximab	Ib/III
	Hepatocellular Carcinoma	NCT06294548	Valemetostat; Atezolizumab; Bevacizumab	Ib/II
	Metastatic Melanoma	NCT04557956	Tazemetostat; Dabrafenib; Trametinib	I/II
CDK7i	Advanced Cancer	NCT05394103	Q901; KEYTRUDA® (pembrolizumab)	I/II
SMARCA2i	Advanced Solid Tumor	NCT05639751	PRT3789; Docetaxel	I
		NCT06682806	PRT3789; pembrolizumab	II

Enhancers are not unique to cancer cells but are co-opted from normal cells [326]. Hence, any therapy that directly targets enhancers could potentially impact the transcriptional mechanisms of normal cells (although cancer cells seem to be more sensitive to this intervention). This may somewhat limit its clinical utility. A potential approach to overcoming this limitation is to precisely deliver drugs to tumor tissues through drug delivery systems, such as a magnetic drug delivery system. This allows for the precise targeting of diseased tissues under the influence of an external magnetic field, thereby reducing peripheral uptake [327].

Metabolic reprogramming supports the growth of tumor cells and has been recognized as a vulnerability to cancer [328]. Combining the targeting of metabolic pathways and enhancers may provide a powerful strategy for improving cancer treatment. Under sufficient oxygen conditions, cancer cells prefer to utilize glycolysis; this preference provides a potential way to distinguish normal cells from tumor cells [329]. Certain key glycolytic enzymes, such as HK, are driven by enhancers and highly expressed in cancer cells [217]. 2-Deoxy-D-glucose (2-DG) is a HK2 inhibitor with a similar structure to that of glucose and has been used as an adjuvant therapy for chemotherapy in various tumors [330]. Recent research has shown that enhancer reprogramming induced by KMT2D deficiency leads to the activation of the glycolytic pathway, which can be inhibited by targeted drugs (e.g., 2-DG). This evidence provides strong support for this therapeutic strategy [16]. Additionally, under MLL3/4 mutation, MLL1 (another member of the COMPASS family) compensates by regulating the expression of purine metabolism-related genes, leading to a significant increase in cellular dependence on purine and pyrimidine metabolism and heightened sensitivity to purine synthesis inhibitors, such as Lometrexol [331]. In conclusion, these findings provide new insights into the connection between epigenetic regulation and metabolic dependencies in cancer, while proposing potential novel strategies for cancer therapy.

Synthetic lethality offers a new possibility for precisely targeting enhancers in cancer cells [332]. Epigenetic modifying enzymes in cancer cells often undergo recurrent mutations that are absent in normal cells. By identifying and targeting these mutated cancer cells, it is possible to achieve specific interference with enhancers [333]. For instance, targeting p300 in cancer cells carrying CBP mutations results in synthetic lethality [334]. The double mutations of SMARCA4 and SMARCA2 have been linked to strong synthetic lethality. When SMARCA2 is mutated, targeting the degradation of SMARCA4 using pharmacological inhibitors (e.g., BRM014) leads to a widespread loss of chromatin accessibility and H3K27ac at enhancer regions [335, 336]. The EP400/TIP60 activity induced by SWI/SNF inhibition is associated with transcriptional recovery; thus, simultaneous ablation of EP400/TIP60 results in a synthetic lethality effect [318]. By utilizing genomic and epigenomic data from patients with cancer, active enhancers, and related synthetic lethal gene pairs can be identified in cancer cells obtained from a specific patient. Based on this information, personalized treatment plans can be designed, selecting the most suitable enhancer inhibitors and other synthetic lethal target inhibitors for combination therapy. In summary, targeting cancer cell enhancers based on the concept of synthetic lethality holds promise for providing an efficient and highly selective strategy for the treatment of cancer.

Antibody-drug conjugate (ADC) drugs provide another powerful way for precisely targeting enhancers. Cancer cells typically express high-specificity and high-abundance antigens on their surface, which are expressed at markedly lower levels in normal cells. This allows ADC drugs to use specific antibodies to recognize and bind to these antigens on the surface of cancer cells. This ensures the delivery of toxic drugs directly into the cancer cells, thereby minimizing damage to normal cells [337]. Additionally, it is possible that ADC drugs can carry traditional chemotherapeutic agents, as well as be combined with epigenetic regulators and metabolic inhibitors, to enhance their anticancer effects. For example, ADC drugs that target enhancer reprogramming can use specific antibodies to deliver epigenetic regulators or metabolic inhibitors precisely to cancer cells, effectively inhibiting cancer cell growth and metastasis. Although the use of ADC drugs has achieved significant clinical success, research on targeting enhancers remains scarce [338]. Future research may focus on optimizing drug carriers, combination therapies, and personalizing ADC treatment plans based on the genomic and epigenomic characteristics of patients and cancer cell surface antigen expression.

Simultaneously, the discovery of high-resolution maps of tumor-infiltrating immune cells has unveiled potential strategies for manipulating the TME. The immunosuppressive nature of TME is, to some extent, driven by enhancer reprogramming, with the potential for reversal [280]. This has been illustrated by recent findings on the interplay between gut microbiota and enhancers. These insights suggest that altering the gut microbiome could modify enhancer activity within the TME, thereby transforming an immunosuppressive environment into one that supports immune-mediated tumor suppression [284]. This emerging understanding opens new avenues for therapeutic intervention, in which the modulation of enhancer landscapes through dietary changes, probiotic supplementation, or targeted microbial therapies(e.g., fecal microbiota transplantation) could become integral components of comprehensive strategies for the treatment of cancer [339, 340].

Additionally, recent advancements have led to the development of an epigenetic modification tool based on CRISPR gene editing technology, termed CRISPRon and CRISPRoff. This tool enables heritable editing of enhancers, paving the way for targeted manipulation of tissue-specific gene expression components and the potential eradication of cancer transcription networks [341]. A recent study utilized CRISPR-Cas9 technology to target the erythroid-specific enhancer of BCL11A, aiming to restore fetal hemoglobin (HbF) expression as a therapeutic strategy for transfusion-dependent β-thalassemia (TDT) and sickle cell disease (SCD). By generating and infusing edited CD34+ hematopoietic stem and progenitor cells (CTX001), both patients demonstrated significantly increased HbF levels and alleviation of disease-related symptoms during follow-up. The TDT patient achieved transfusion independence, while the SCD patient experienced no recurrence of vaso-occlusive crises. These findings suggest that enhancer-targeted gene editing offers an effective and durable treatment approach for these hemoglobinopathies.

Despite the promising potential of enhancer-targeting therapies, several challenges remain unresolved. One significant challenge is the emergence of acquired resistance. For instance, the dual EZH1/EZH2 inhibitor valemetostat initially shows strong therapeutic efficacy; however, resistant clones often emerge with prolonged treatment. This resistance may be driven by mutations in key subunits of the PRC2 complex, such as EZH2 or EED, which reduce the inhibitory efficiency of valemetostat and restore H3K27me3 levels. Additionally, resistance may develop through compensatory mechanisms involving DNA methylation. Tumor cells can upregulate DNMT3A or lose TET2 function, substituting DNA methylation for H3K27me3 marks to re-silence critical tumor suppressor genes and restore a malignant phenotype [314]. These mechanisms suggest that monotherapy targeting enhancers may be insufficient to overcome the epigenetic adaptability of enhancer-dependent cancers.

Another major challenge is the highly dynamic nature of enhancer function, which can vary significantly across cell types, developmental stages, and disease states. This heterogeneity arises not only from genetic background differences but also from multiple layers of regulation, including chromatin state, epigenetic marks, and transcription factor binding dynamics. Single-cell ATAC and CUT&Tag studies have revealed substantial variation in enhancer accessibility and epigenetic modifications across different clones, directly impacting the expression of key genes and functional differentiation of tumors [68, 280, 342, 343]. For example, single-cell ATAC sequencing has shown differences in enhancer networks within tumor-infiltrating immune cells that influence therapeutic responses [342], while single-cell CUT&Tag has clarified how H3K27me3 marks are redistributed in different clones to repress or activate specific genes [68]. This heterogeneity may result in inconsistent therapeutic outcomes among patient groups and contribute to the emergence of treatment resistance due to dynamic tumor adaptation [68].

During tumor progression, enhancers can undergo reprogramming, activating or repressing specific gene networks through transcription factor reassembly or chromatin state alterations, thereby driving phenotypic plasticity to adapt to new microenvironments or therapeutic pressures [155]. For example, in pancreatic cancer, PRMT1 modulates the methylation state of enhancers to restrict gemcitabine-induced transcription factor binding (e.g., MAFF and BACH1), reprogramming enhancer activity and promoting chemotherapy resistance [344].

Although epigenomics and single-cell technologies have provided critical insights into the dynamic changes in enhancer activity, their clinical application is hindered by high costs, technical complexity, and time consumption. Real-time monitoring of enhancer activity changes in patients and correlating these with therapeutic outcomes remains a significant technical and logistical challenge.

These challenges underscore the need for innovative strategies and tools to enhance the efficacy and specificity of enhancer-targeting therapies while addressing resistance and heterogeneity in cancer treatment.

## Conclusion and prospect

Enhancer reprogramming constitutes a fundamental mechanism underpinning the transcriptional dysregulation in cancer, influencing cell fate decisions, and contributing to the phenotypic plasticity of cancer cells [98]. The intricate interaction between enhancers and the transcriptional machinery dictates the cellular identity and functionality, rendering enhancers critical determinants of cancer progression and viable targets for therapeutic intervention [94]. The reprogramming of enhancers in cancer is a double-edged sword, driving oncogenesis while offering a strategic target for disrupting the aberrant transcriptional networks that sustain cancer cells. The therapeutic potential of targeting enhancer dysregulation holds promise for the development of innovative cancer treatments. This approach offers a path to disrupt the addiction of cancer cells to aberrant enhancer-driven transcriptional programs. Enhancer-driven transcription networks are frequently remodeled during cancer resistance and metastasis, providing an opportunity to predict patient prognosis, while showing potential advantages in assessing the likelihood of metastasis [148, 301].

Despite this promise, targeting enhancers for therapy presents significant challenges that need to be addressed. Enhancers are often tissue- and cell-type-specific, which increases the difficulty of achieving specificity without off-target effects [54]. Off-target activity could disrupt normal transcription programs in healthy cells, leading to unintended consequences [22, 345, 346]. Furthermore, cancer cells may develop resistance to enhancer-targeting therapies, either through the rewiring of transcriptional networks or the activation of alternative enhancers, which necessitates a deeper understanding of resistance mechanisms and the development of strategies to overcome them [347].

Due to the lack of drugs specifically targeting cancer cell enhancers, future research may focus more on precision therapy strategies, such as those based on the concept of synthetic lethality or the use of ADC drugs [348, 349]. Additionally, the abnormal metabolic pathways exhibited by cancer cells also present a potential vulnerability. Synthetic biology techniques have also been utilized to design and construct artificial enhancers for the precise regulation of gene expression and the development of novel gene therapy strategies. However, these approaches face technical and biological limitations, including achieving spatial and temporal control of enhancer activity and avoiding unintended activation of oncogenic programs.

Although it is established that enhancers exhibit different activities in various tissues and cell types, the specific underlying molecular mechanisms remain unclear. For example, the factors that determine the activity of an enhancer in a particular tissue remain unknown. This is significant in deciphering the abnormal activity of enhancers in cancer. For instance, the circumstances under which FOXA1 is activated, leading to liver metastasis of pancreatic cancer, are also unknown [148]. Current research mainly focuses on aberrantly activated enhancers; there is limited knowledge regarding enhancers that are silenced in cancer. Understanding this mechanism could help researchers manipulate those silenced enhancers to reactivate normal transcription programs. Despite the known importance of TFs in controlling enhancer activity, the mechanisms by which they are repositioned on enhancers remain unexplained. Further investigation into enhancer specificity, resistance mechanisms, and their interplay with the broader transcriptional network will enrich our understanding of enhancers and provide new perspectives and methods for gene regulation, biological research, and clinical applications.
